# Advancements in Bio‐Integrated Flexible Electronics for Hemodynamic Monitoring in Cardiovascular Healthcare

**DOI:** 10.1002/advs.202415215

**Published:** 2025-04-25

**Authors:** Ke Huang, Zhiqiang Ma, Bee Luan Khoo

**Affiliations:** ^1^ Department of Biomedical Engineering City University of Hong Kong Hong Kong 999077 China; ^2^ Hong Kong Center for Cerebro‐Cardiovascular Health Engineering (COCHE) Hong Kong 999077 China; ^3^ Department of Precision Diagnostic and Therapeutic Technology City University of Hong Kong Shenzhen‐Futian Research Institute Shenzhen 518057 China

**Keywords:** artificial intelligence, bio‐integrated electronics, cardiovascular diseases, flexible conformal sensors, hemodynamic monitoring

## Abstract

Cardiovascular diseases (CVDs) remain the leading cause of global mortality, highlighting the urgent need for effective monitoring and prevention strategies. The rapid advancement of flexible sensing technology and the development of conformal sensors have attracted significant attention due to their potential for continuous, real‐time assessment of cardiovascular health over extended periods. This review outlines recent advancements in bio‐integrated flexible electronics designed for hemodynamic monitoring and broader CVD healthcare applications. It introduces key physiological indicators relevant to hemodynamics, including heart rate, blood pressure, blood flow velocity, and cardiac output. Next, it discusses flexible bio‐integrated electronics engineering strategies, such as working principles and configuration designs. Various non‐invasive and invasive bio‐integrated devices for monitoring these hemodynamic indicators are then presented. Additionally, the review highlights the role of artificial intelligence algorithms and their practical applications in bio‐integrated electronics for hemodynamic detection. Finally, it proposes future directions and addresses potential challenges in the field.

## Graphical abstract

This review explores the cutting‐edge development of bio‐integrated flexible electronics for real‐time hemodynamic monitoring in cardiovascular healthcare. It covers key physiological indicators, innovative sensing mechanisms, and materials considerations. This paper highlights the application of both invasive and non‐invasive devices in cardiovascular monitoring discusses the integration of AI to enhance signal processing, and outlines future research directions in the field.

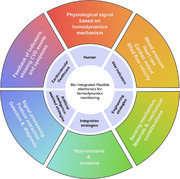

## 1 Introduction

As populations age and lifestyles evolve, cardiovascular diseases (CVDs) have become a leading global cause of mortality, accounting for over 17 million deaths annually and representing one‐third of all global fatalities.^[^ ^1^ ^]^ Hemodynamic monitoring is increasingly important in evaluating cardiovascular health and managing patient care. Traditionally, clinical methods such as sphygmomanometers,^[^ ^2^ ^]^ Holter monitors,^[^ ^3^ ^]^ Echocardiography,^[^ ^4^ ^]^ and magnetic resonance imaging (MRI)^[^ ^5^ ^]^ have been employed to obtain hemodynamic information. However, these approaches face significant limitations regarding real‐time capabilities and portability, which hinder the pursuit of remote and intelligent healthcare solutions. Many conventional monitoring methods rely on intermittent measurements that may miss acute changes in hemodynamic indices, potentially leading to delayed interventions in critical situations.^[^ ^6^, ^7^ ^]^

With the rapid advancement of microelectromechanical systems (MEMS) technology, various wearable devices for hemodynamic monitoring have been developed, including electronic blood pressure monitors, smart bracelets, and smartwatches.^[^ ^8^ ^]^ While these innovations facilitate mobile and home‐based hemodynamic monitoring, they face two significant challenges that impede broader adoption: 1) limited integration and comfort, as most wearable devices require external fixation to body parts (e.g., the wrist), which significantly reduces user comfort;^[^ ^9^ ^]^ 2) Limited monitoring accuracy, as the majority of these products have not undergone clinical validation to meet established diagnostic standards.^[^ ^10^ ^]^

Fortunately, emerging flexible electronics offer practical solutions for intelligent healthcare by enabling continuous data acquisition, capturing dynamic changes in physiological parameters, and providing clinical‐level high sensitivity for detecting minute deformations in arterial walls. These devices can monitor multiple hemodynamic indices simultaneously while ensuring high comfort and adaptability to the skin, making them suitable for long‐term wear. Their lightweight and conformable designs allow patients to maintain a high activity level without feeling restricted by monitoring equipment.^[^ ^11^, ^12^, ^13^, ^14^ ^]^ By leveraging advanced materials and technologies,^[^ ^15^, ^16^ ^]^ researchers have developed a range of flexible bio‐integrated electronics capable of precisely decoding hemodynamic information, such as heart rate (HR), blood pressure (BP), cardiac output (CO), and blood flow velocity (BFV).^[^ ^17^, ^18^, ^19^, ^20^ ^]^ These devices demonstrate significant potential for early cardiovascular disease screening, rehabilitation monitoring, and personalized health management.^[^ ^21^ ^]^ Moreover, integrating artificial intelligence enhances precise and remote healthcare delivery capacity.^[^ ^22^, ^23^ ^]^

**Figure 1:**
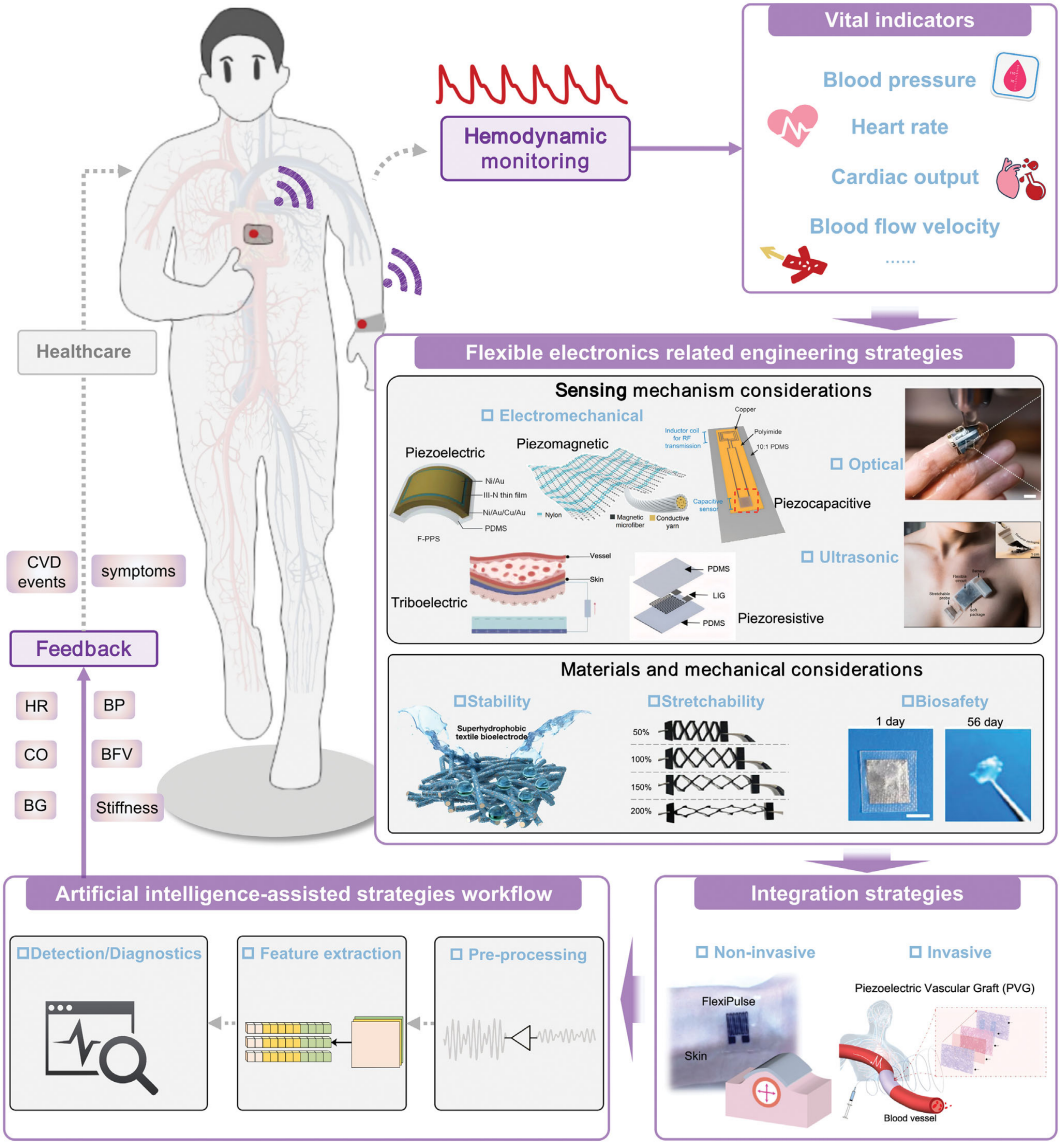
Overall view of bio‐integrated flexible electronics for hemodynamics monitoring and management. Reproduced with permission.^[^ ^11^ ^]^ Copyright 2023, Elsevier. Reproduced with permission.^[^ ^122^ ^]^ Copyright 2024, American Association for the Advancement of Science. Reproduced with permission.^[^ ^137^ ^]^ Copyright 2022, Elsevier Ltd. Reproduced with permission.^[^ ^145^ ^]^ Copyright 2025, Wiley‐VCH GmbH. Reproduced with permission.^[^ ^146^ ^]^ Copyright 2025, WILEY‐VCH GmbH. Reproduced with permission.^[^ ^147^ ^]^ Copyright 2024, WILEY‐VCH. Reproduced with permission.^[^ ^169^ ^]^ Copyright 2021, Elsevier. Reproduced with permission.^[^ ^176^ ^]^ Copyright 2019, WILEY‐VCH GmbH. Reproduced with permission.^[^ ^77^ ^]^ Copyright 2021, WILEY‐VCH GmbH. Reproduced with permission.^[^ ^180^ ^]^ Copyright 2021, Springer Nature. Reproduced with permission.^[^ ^182^ ^]^ Copyright 2024, Springer Nature.

This study summarizes recent advances in bio‐integrated flexible electronics for continuously monitoring hemodynamics. An overview of the study is illustrated in **Figure** **1**.

Initially, we present the fundamental mechanisms of hemodynamics, discussing its four major cardiovascular vital indicators: heart rate (HR), blood pressure (BP), blood flow velocity (BFV), and cardiac output (CO). We then outline the engineering strategies for developing bio‐integrated flexible electronics, including typical working mechanisms, materials, and mechanical considerations. Subsequently, we describe representative bio‐integrated flexible electronics for hemodynamic monitoring, focusing on invasive and non‐invasive approaches. Furthermore, the application of artificial intelligence in signal processing for intelligent hemodynamic monitoring is examined, covering basic algorithms such as data preprocessing and machine/deep learning, as well as typical demonstrations, including BP evaluation and cardiovascular disease diagnostics. Finally, we discuss perspectives and future directions for developing flexible bio‐integrated electronics in hemodynamic monitoring.

## 2 Biological Mechanism of Hemodynamics

### 2.1 Fundamentals of Hemodynamics and Relevance to Flexible Electronics

Hemodynamics studies the forces and mechanics involved in blood circulation within the human body, providing critical insights into cardiovascular function and health. It encompasses the intricate interplay among the pumping action of the heart, the vascular system, and the dynamic properties of blood. This foundational understanding is vital for developing advanced monitoring technologies, including bio‐integrated flexible electronics, which aim to provide continuous, accurate, and non‐invasive hemodynamic assessments.

At its core, hemodynamics relies on fluid mechanics principles, but it introduces unique complexities due to the vascular system's pulsatile, flexible, and dynamic nature. Unlike traditional fluid dynamics, blood flow occurs within a network of compliant, branching vessels, adapting to changes in pressure, resistance, and flow velocity. Adding to these complexities, blood is a living tissue, with non‐Newtonian properties and the ability to transport oxygen, nutrients, hormones, and waste products throughout the body.^[^ ^24^ ^]^ These characteristics make accurate hemodynamic monitoring both scientifically and technically challenging.

The heart is the central pump driving the circulatory system, generating rhythmic contractions that propel blood through arteries, capillaries, and veins.^[^ ^25^, ^26^ ^]^ This process ensures adequate perfusion to tissues and organs. Systemic circulation delivers oxygenated blood and nutrients to the body, while pulmonary circulation facilitates gas exchange in the lungs. The vascular system—a complex network comprising arteries, veins, and capillaries—is crucial in maintaining hemodynamic balance. Arteries, with their elastic and muscular walls, withstand high pressures and deliver oxygenated blood to tissues, while veins, operating at lower pressures, return deoxygenated blood to the heart.^[^ ^27^ ^]^ As the smallest blood vessels, capillaries mediate the exchange of oxygen, nutrients, and metabolic waste between blood and surrounding tissues.^[^ ^28^, ^29^ ^]^ Key hemodynamic parameters include blood flow velocity, pressure, and resistance, which vary across the vascular system depending on vessel diameter, elasticity, and branching architecture. These parameters are critical for maintaining homeostasis and ensuring proper nutrient and oxygen delivery.

Traditional hemodynamic measurement techniques, such as sphygmomanometry and catheter‐based systems, have provided valuable insights but are often limited by invasiveness, intermittent data collection, or lack of wearability. Therefore, the unique physiological challenges of hemodynamics underscore the importance of advanced monitoring technologies. Flexible bio‐integrated electronics offer a transformative solution for hemodynamic monitoring, blending seamless integration with the body, high sensitivity, and the capability for continuous, long‐term data capture. These systems leverage soft, lightweight, and stretchable materials to conform to the skin or vascular surfaces, enabling non‐invasive or minimally invasive monitoring of critical hemodynamic parameters.^[^ ^30^, ^31^ ^]^

Integrating hemodynamic principles with flexible electronics highlights the importance of understanding blood flow mechanics and associated parameters for device design. For instance, flexible pressure sensors based on piezoresistive or piezoelectric mechanisms can measure blood pressure dynamically by detecting subtle changes in arterial wall deformation. Similarly, optical and ultrasonic flexible sensors can change deep skin blood vessels dynamically. These techniques allow high accuracy in the real‐time monitoring of cardiovascular health indicators, offering significant potential for early disease detection and personalized healthcare. Moreover, flexible electronics can be designed to monitor the pulsatile nature of blood flow, capturing waveform data that reflect heart function and vascular compliance. By analyzing these signals, clinicians can assess systemic hemodynamic behavior and detect abnormalities such as hypertension, atherosclerosis, or heart failure. These technologies also enable continuous monitoring during physical movement or over extended periods, providing a more comprehensive picture of a patient's cardiovascular health than traditional, snapshot‐based methods.

A thorough understanding of hemodynamics provides the foundation for designing flexible electronics capable of monitoring cardiovascular health. The interplay between hemodynamic principles and sensor technology ensures accurate and robust measurement and facilitates the development of next‐generation diagnostic and therapeutic tools. By bridging the gap between biology and technology, flexible electronics hold immense potential to revolutionize hemodynamic monitoring and advance cardiovascular medicine.

### 2.2 Crucial Indicators of Hemodynamic Monitoring

In clinical practice, hemodynamic monitoring provides a comprehensive view of the circulatory system, encompassing vital parameters that illuminate cardiac pumping efficiency and vascular functionality, thereby facilitating the diagnosis and treatment of circulatory disorders.^[^ ^32^ ^]^ Through meticulous hemodynamic surveillance, healthcare professionals can develop personalized treatment regimens tailored to individual patients’ physiological nuances and disease characteristics.^[^ ^33^ ^]^ Furthermore, hemodynamic monitoring is an essential tool in the care of critically ill patients, enabling the prompt detection and management of systemic circulation anomalies while minimizing complications to optimize clinical outcomes.^[^ ^34^ ^]^

**Figure 2:**
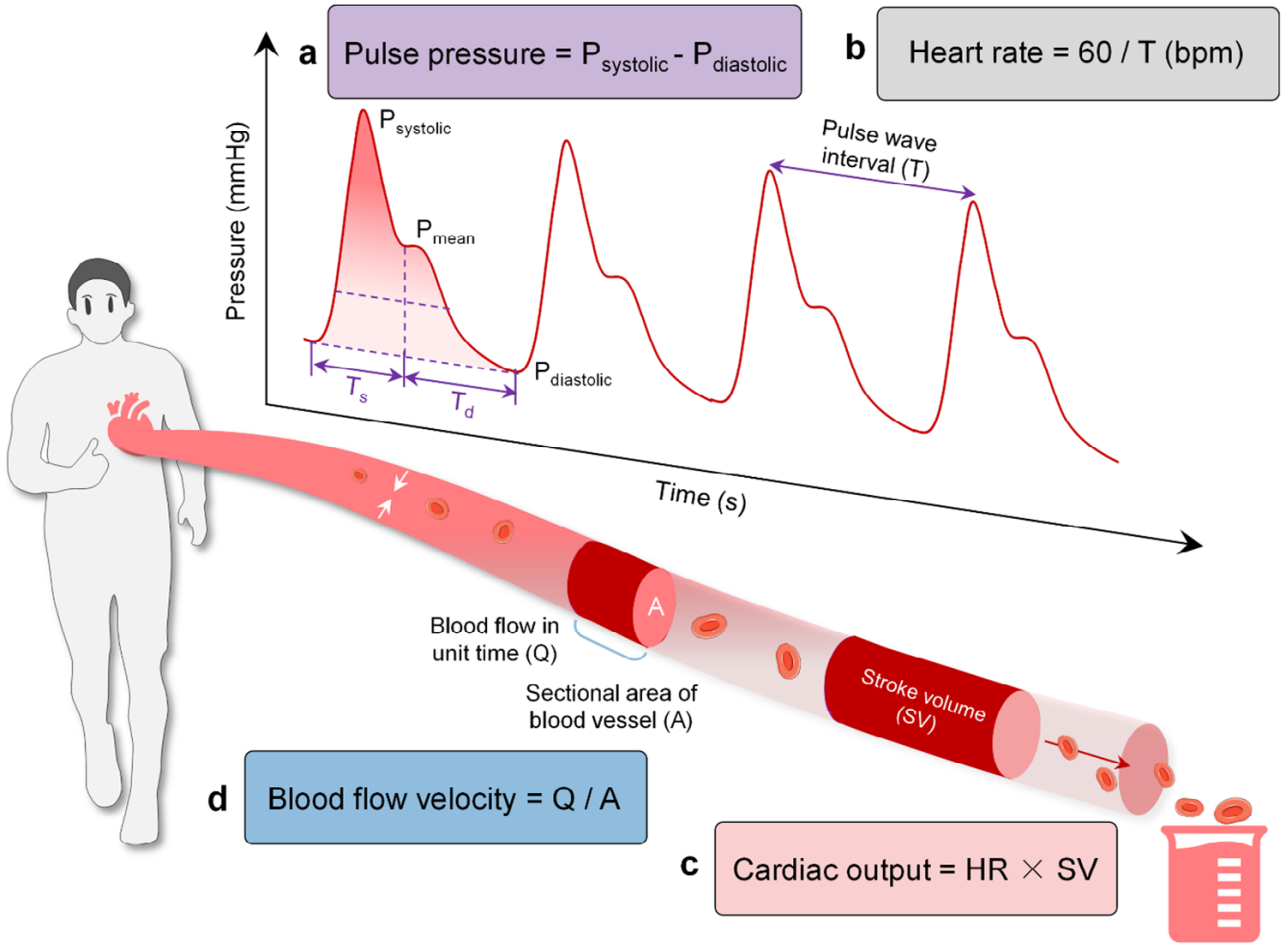
Typical indicators for hemodynamic monitoring. a) Blood pressure (unit: mmHg) and pulse waveform correspond to systolic and diastolic blood pressure. b) Heart rate, the number of heart beats per minute (bpm) c) Cardiac Output, the volume of blood ejected by the heart (stroke volume) per minute. d). Blood flow velocity is the rate at which blood traverses through the vascular system.

To perform effective and precise monitoring, selecting and quantifying key indicators from hemodynamics is imperative. Extensive research has identified four primary physiological indicators—blood pressure (BP), heart rate (HR), blood flow velocity (BFV), and cardiac output (CO)—as essential for accurate hemodynamic monitoring, as illustrated in **Figure** **2**. Each indicator offers a unique perspective on hemodynamic and cardiovascular status, providing insights into the heart's pumping efficiency, blood flow through the vessels, and overall system health. Clinicians closely monitor these vital signs to detect anomalies that may signal disease onset or require therapeutic adjustments. By understanding these indicators, healthcare professionals can navigate the complexities of cardiovascular care more effectively. At the same time, engineers can develop more advanced bio‐integrated electronics for cardiovascular healthcare, ensuring the sustained vitality of this essential system.

#### 2.2.1 Blood Pressure

Blood pressure (BP) refers to the lateral force exerted by blood on the walls of blood vessels per unit area. As blood circulates through the vascular system, it exerts oscillatory pressure on the vessel walls, influenced by the heart's pumping action.^[^ ^35^ ^]^ This pressure consists of several components: systolic blood pressure (SBP), which represents the maximum pressure in the arterial wall; diastolic blood pressure (DBP), indicating the minimum pressure in the arterial wall; and mean blood pressure (MBP), which reflects the average pressure within the arteries, as illustrated in Figure 2a. **Table** **1** presents the range of BP values associated with cardiovascular health, as defined by the World Health Organization (WHO) in 2021.^[^ ^36^ ^]^

**Table 1:** The range of values corresponding to blood pressure classification categories.

Blood pressure classification category	Diastolic [mmHg]	Systolic [mmHg]
Normal	≤ 80	≤ 120
High	≥90	≥140
Grade 1 hypertension	90–99	140–159
Grade 2 hypertension	100–109	160–179
Grade 3 hypertension	≥110	≥180

BP values closely reflect an individual's cardiovascular status and overall health. Elevated pulse pressure is often associated with aortic sclerosis, aortic insufficiency, arteriovenous fistula, and hyperthyroidism.^[^ ^37^ ^]^ Conversely, reduced pulse pressure is typically observed in pericardial effusion, constrictive pericarditis, and peripheral circulatory failure.^[^ ^38^ ^]^

Accurate assessment of BP is critical for cardiovascular monitoring and healthcare. Unlike traditional clinical BP evaluation devices, such as cuff‐type BP gauges, emerging flexible skin‐integrated BP sensors offer significant advantages, including lightweight design, enhanced comfort, high accuracy, and real‐time and long‐term monitoring capability. The most used method for BP evaluation with flexible skin‐integrated sensors is based on pulse wave velocity (PWV), calculated using the Moens‐Korteweg equation,^[^ ^39^ ^]^ which is expressed as follows:

(1) $$PWV=K\sqrt{\frac{Eh}{\rho d}}$$

where *K* stands for the Moens constant (*K* = 0.8 when applied to the human aorta), *E* is the elastic modulus of the artery, *h* is the thickness of the artery wall, *d* is the artery's diameter, and ρ is blood density. In 1933, the Moens‐Kortweg equation was modified by Bramwell and Hill, as below:^[^ ^40^ ^]^

(2) $$PWV=\sqrt{\frac{V*dP}{\rho *dV}}=\sqrt{\frac{r*dP}{\rho *2*dr}}\;$$

where *P* is pressure, and is the volume per unit length *V*, ρ is the blood density, and *r* is the radius of the artery. Specifically, a curvilinear relationship exists between arterial pressure and volume, whereby arterial compliance (*V/P*) decreases as pressure increases while the volume increases due to arterial dilation. This phenomenon directly contributes to increased PWV. In addition, the time it takes for the arterial pulse wave to propagate between two points along an artery is measured as a vital indicator, which is typically done by placing sensors at two points along the artery and then measuring the time delay between the arrival of the pulse wave at each sensor. The distance between the two sensors is also measured, and the PWV can then be calculated using the following formula:

(3) $$PWV=\frac{L}{PPT}\;$$

where *L* is the interval distance between sensors, and *PPT* is the time delay between pulse wave arrival at each sensor. Once the PWV is determined, it can be used to estimate BP using an equation that relates PWV to BP and a reference values equation that depends on factors such as age, gender, and other health conditions.

To estimate sustained blood pressure, bioelectrical, optoelectronic, and electromechanical sensors are conventionally utilized for signal acquisition.^[^ ^35^, ^41^, ^42^, ^43^ ^]^ These signals undergo algorithmic processing, employing metrics such as pulse transit time (PTT) or pulse arrival time (PAT) in conjunction with machine learning (ML) algorithms to enhance precision and accuracy.^[^ ^44^, ^45^ ^]^

Before deployment in clinical settings, flexible skin‐integrated BP sensors must achieve clinical accuracy. Two primary certification standards govern the precision of international BP monitors. The first pertains to electronic or aneroid BP monitors, as defined by the Association for the Advancement of Medical Instrumentation (AAMI) and its corresponding accuracy evaluation program.^[^ ^46^, ^47^ ^]^ The second standard was introduced by the British Society of Hypertension in 1990. Notably, the average discrepancy compared to mercury sphygmomanometers does not exceed 5 mmHg, confirming that the accuracy of electronic sphygmomanometers aligns with established international benchmarks.^[^ ^48^ ^]^

#### 2.2.2 Heart Rate

As a pivotal indicator in assessing cardiovascular system health and functionality, heart rate (HR) is quantified as the number of heart beats per minute (bpm), which can be calculated from (Figure 2b):

(4) $$HR=\frac{60}{T}\;\left(bpm\right)$$

where *T* signifies the duration of an entire cardiac cycle.

Alternatively, employing Fast Fourier Transform (FFT) analysis provides an effective method for assessing HR.^[^ ^49^, ^50^ ^]^ Typical HR for adult humans generally falls within 60 to 100 *bpm*.^[^ ^51^ ^]^ However, individual variations exist; athletes and individuals in excellent physical condition may exhibit a lower resting HR, while certain health conditions or specific medications can result in an elevated HR.^[^ ^52^ ^]^ Persistently elevated or diminished HR may indicate underlying cardiovascular issues, metabolic disorders, or thyroid abnormalities, among other potential health concerns.^[^ ^53^ ^]^ Therefore, HR monitoring is invaluable for evaluating cardiovascular health.

HR can be obtained from pulse waveforms detected through various techniques, including electrocardiography (ECG),^[^ ^54^ ^]^ photoplethysmography (PPG),^[^ ^55^ ^]^ seismocardiography (SCG),^[^ ^56^ ^]^ and ballistocardiography (BCG).^[^ ^57^ ^]^ The International Organization for Standardization (ISO 60601‐2‐25), the Association for the Advancement of Medical Instrumentation (AAMI EC13), and the U.S. Food and Drug Administration (FDA 510(k) Premarket Submission) have established stringent standards and guidelines governing the clinical accuracy of HR monitoring devices.^[^ ^58^ ^]^ These devices must demonstrate a direct correlation and high level of consistency with established benchmark methods, such as electrocardiography, to ensure their reliability and accuracy in clinical settings.

#### 2.2.3 Cardiac Output

Cardiac output (CO) is defined as the volume of blood ejected per minute by either the left or right ventricle. As illustrated in Figure 2c, CO quantifies the amount of blood pumped by the heart within a specified timeframe,^[^ ^59^ ^]^ reflecting the heart's ability to supply oxygen‐rich blood to the body's tissues. In arterial waveform analysis,^[^ ^60^ ^]^ CO can be derived by examining total peripheral resistance and mean arterial pressure, as demonstrated below:

(5) $$CO=\frac{P_{mean}}{TPR}\;$$

where *P_mean_* denotes mean arterial pressure, and *TPR* denotes total peripheral resistances.

In 1970, Kochoukos et al. introduced a more accurate method for estimating heart stroke volume (SV) by measuring the area under the systolic portion of the arterial pressure waveform.^[^ ^61^ ^]^ Subsequently, in 1989, Wesseling et al. developed an algorithm (Equation 7) for calculating SV based on aortic impedance and the changes in arterial pressure during systole,^[^ ^62^, ^63^ ^]^ which then CO can be calculated as

(6) CO=SV×HR

where the estimation of *SV* is dependent on the integral of the change in pressure *P* from end diastole to end systole (*t*) and the impedance *Z* of the aorta:

(7) $$SV=\frac{\int dP/dt}{Z}\;$$

However, variations in total peripheral resistance can affect the accuracy of these measurements. A primary challenge in analyzing arterial waveforms is that aortic impedance is influenced by both cardiac output and aortic compliance.^[^ ^64^ ^]^ Consequently, estimating SV changes rather than obtaining absolute values reliably is more feasible; prior calibration of such systems is essential before their application.

Additionally, Doppler echocardiography utilizes ultrasonic probes to measure the blood flow velocity of the heart and the cross‐sectional area of the blood vessels, subsequently calculating CO based on these parameters.^[^ ^65^ ^]^ Furthermore, CO can be assessed by measuring changes in the resistance or impedance of body tissues to an electrical current, with electrodes placed on the chest and back.^[^ ^66^ ^]^

In humans, CO typically ranges from 5 to 6 L min^−1^ at rest, increasing to more than 35 L min^−1^ in elite athletes during exercise.^[^ ^67^ ^]^ Abnormal CO values may indicate a variety of underlying health conditions. For instance, elevated CO can be associated with anemia, hyperthyroidism, fever, or infections. Conversely, conditions such as heart failure, arrhythmias, myocardial damage, or reduced blood volume can result in CO levels that fall below the normal range. Prolonged abnormal CO levels may present with a range of symptoms, including fatigue, respiratory difficulties, lethargy, palpitations, and dizziness.^[^ ^68^, ^69^, ^70^ ^]^

#### 2.2.4 Blood Flow Velocity

Blood flow velocity refers to the rate at which blood moves through the vascular system. Several key factors influence this velocity, including cardiac pumping capacity, vascular resistance, blood viscosity, and vessel diameter.^[^ ^71^ ^]^ Blood flow velocity can be calculated using the following formula:

(8) $$v=\frac{Q}{A}$$

where *v* represents blood flow velocity (unit: cm s^−1^ or m s^−1^), *Q* represents blood flow (unit: mL s^−1^ or L min^−1^), and *A* denotes the cross‐sectional area of the vessel (unit: cm^2^ or m^2^), as illustrated in Figure 2d.

The standard range of blood flow velocity varies among individuals and is influenced by several factors, including age, gender, body composition, and the specific examination site. Smaller vessels exhibit higher blood flow velocities, whereas larger vessels generally experience slower flow rates.^[^ ^72^ ^]^ Various diseases can lead to alterations in blood flow velocity. For instance, increased resistance within constricted vessels often results in a reduction of flow rate.^[^ ^73^ ^]^ Conditions such as atherosclerosis and thrombosis contribute to vessel narrowing and rigidity. Additionally, sustained high BP can increase arterial stiffness.^[^ ^74^ ^]^ In heart failure, the heart's ability to pump blood effectively diminishes, while venous insufficiency may lead to blood pooling in the veins, further contributing to decreased blood flow velocity.^[^ ^75^ ^]^

Monitoring hemodynamic indices—such as BP, HR, CO, and pulse flow velocity—is essential for disease monitoring and management, such as heart failure and hypertension. Flexible electronics have clear advantages over conventional wearables for hemodynamic monitoring. Clinically, hemodynamics is usually monitored using catheterization and oscillometry. Catheterization is invasive with a high risk of infection, and oscillometry provides only intermittent data, which may lead to missed critical changes in a patient's condition. They are uncomfortable and require specialized training. Compared to traditional wearable products, flexible electronic devices are often designed to be lighter and thinner, making them more comfortable, able to fit the skin better, and less disruptive to the user's daily activities. In addition, flexible electronics are highly integrated. They can incorporate multiple sensor functions in a small device, enabling it to simultaneously monitor various physiological indicators, such as HR, BP, and pulse wave flow. Specific flexible sensors can capture hemodynamic indicators in real time and perform data analysis and feedback through a smartphone application, which provides richer information than traditional wearable devices.^[^ ^76^, ^77^ ^]^ Their portability allows flexible electronics to be used in various settings, including at home. This is especially beneficial for patients with chronic conditions who need regular monitoring, as traditional devices often require frequent medical visits. Flexible electronics also enable advanced data processing and wireless connectivity, allowing real‐time data sharing with healthcare providers. This facilitates timely clinical decisions and personalized feedback.

This motivates researchers to develop various bio‐integrated flexible electronics for hemodynamic monitoring. In recent years, advancements in bio‐integrated flexible electronics have achieved clinical detection accuracy thanks to suitable sensing principles and practical material and structural design. These innovations have begun to undergo clinical validation and are being explored to diagnose related diseases. Among them, some of the most effective sensors and systems have reached clinical accuracy and realized a variety of clinical diseases such as arrhythmia and atrial septal defect.^[^ ^11^, ^16^, ^78^ ^]^

## 3 Engineering Strategies for Bio‐Integrated Flexible Electronics Development

### 3.1 Sensing Mechanism Consideration

Flexible bio‐integrated electronics hold significant potential in medical technology, particularly for monitoring cardiovascular hemodynamics. These devices are distinguished by their ability to integrate seamlessly with soft biological tissues, enabling continuous, high‐quality signal acquisition while allowing extended observation periods without discomfort. Driven by this capability, researchers have developed a range of flexible bio‐integrated electronics specifically for hemodynamic monitoring. Based on their working principles, the three most used types are involved: electromechanical (including piezoresistive, piezoelectric, triboelectric, piezocapacitive, and piezomagnetic), optical, and ultrasonic.

**Figure 3:**
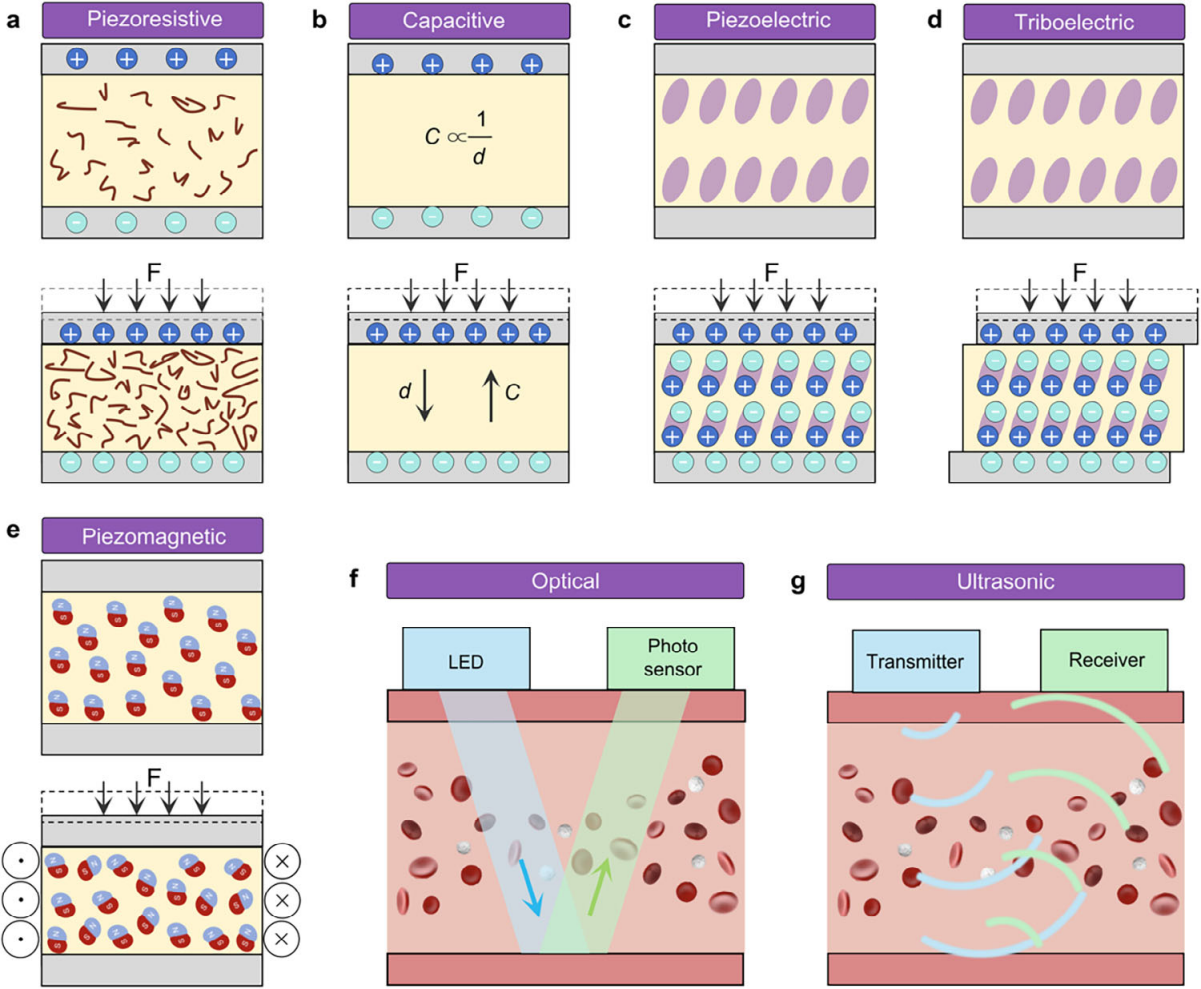
Transduction principles of seven common sensing mechanisms. a) The piezoresistive mechanism converts pressure into resistance change, b) capacitive mechanism converts pressure into capacitive change, c) piezoelectric mechanism converts pressure into voltage change, d) the triboelectric mechanism converts pressure into charge change, e) piezomagnetic mechanism converts pressure into magnetic flux density change, f) optical mechanism converts blood flow into gratings displacement change, and g) ultrasonic mechanism converts blood flow into doppler frequency shift.

In the design of flexible bio‐integrated electronics, the selection of the sensing mechanism is a critical consideration. Several factors influence this choice, including energy efficiency, degree of integration, and working environment. For example, flexible electronics utilizing piezoelectric or triboelectric mechanisms are self‐powered, operating without an external power supply, enhancing user convenience and promoting device sustainability. Piezoresistive‐based flexible electronics offer easy integration and strong electromagnetic interference resistance, making them suitable for environments with significant electromagnetic activity. Additionally, ultrasonic flexible electronics can penetrate deep tissues, allowing for the monitoring of physiological signals from within the body. To facilitate a better understanding and selection of these mechanisms, we will discuss the fundamental working principles and characteristics of seven types of sensing mechanisms in this section, as illustrated in **Figure** **3**.

#### 3.1.1 Electromechanical Working Principles

The dynamics of blood flow, governed by vasoconstriction and vasodilation, influence blood volume and the pulsatile pressure patterns within blood vessels. Vasoconstriction, which tightens and narrows arterial vessels, enhances blood circulation, and increases arterial pressure, while vasodilation relaxes and widens these vessels, reducing arterial pressure.^[^ ^79^ ^]^ These physiological processes generate mechanical deformations in the circulation, which can be reflected in the skin strain above the artery and the heart's pumping action. Electromechanical sensors, particularly strain sensors, can detect variations in mechanical strain at the surface, encompassing piezoresistive, capacitive, piezoelectric, triboelectric, and magnetic types.^[^ ^80^, ^81^, ^82^ ^]^ This section details these mechanisms, highlighting their unique contributions to the precise and responsive monitoring of hemodynamics.

##### Piezoresistive Sensing Mechanism

The piezoresistive effect can transfer mechanical stimuli into electrical resistance variations, as shown in Figure 3a. These variations in resistance correlate directly with the magnitude of the applied external pressure, a relationship that can be expressed by

(9) $$R=R_{0}+S\times P$$

where *R* is the resistance value of the sensor, *R_0_* is the initial resistance without mechanical stimulus, *S* is the sensor's sensitivity, indicating the change rate of the resistance value relative to the pressure, and *P* is the pressure applied to the sensor. The alteration in electrical resistance is typically detected and quantified using a classical bridge circuit.^[^ ^83^ ^]^ This circuit outputs voltage signals directly proportional to the applied external pressure. The resulting signal is then transmitted to amplifier circuits for further amplification and precise measurement.

Piezoresistive sensors present a cost‐effective and readily accessible option for production, offering several advantages, including ease of acquisition and utilization, commendable durability, seismic resilience, and rapid response times.^[^ ^84^ ^]^ Various functional materials are suitable for developing piezoresistive sensors, including semiconductor materials,^[^ ^85^ ^]^ conductive nanoparticle‐based composites,^[^ ^86^, ^87^, ^88^, ^89^ ^]^ and metal films.^[^ ^90^, ^91^ ^]^ However, piezoresistive sensors also encounter certain limitations in practical applications. For instance, the precision and linearity of these sensors may be affected by environmental temperature and humidity fluctuations, potentially leading to measurement discrepancies.^[^ ^92^ ^]^

##### Piezocapacitive Sensing Mechanism

Piezocapacitive sensors convert mechanical stimuli into variations in electrical capacitance, as illustrated in Figure 3b. A typical configuration of a piezocapacitive sensor comprises an insulating layer sandwiched between two electrodes.^[^ ^93^ ^]^ The fundamental capacitance function can be expressed as follows:

(10) $$C=\frac{\varepsilon_{0}\varepsilon_{r}A}{d}\;$$

where *C* is the capacitance value (in *farads*, *F*), *ε_0_* is the dielectric constant of vacuum (about 8.854 × 10⁻¹^2^ F/m), *ε_r_* is the relative dielectric constant of the medium, *A* is the effective area between the capacitor plates, and *d* is the distance between the capacitor plates. Blood flows through a vessel, generating minute vibrations at the skin's surface.^[^ ^94^ ^]^ These mechanical strains alter the spacing between the two electrodes in a piezocapacitive sensor, producing capacitive signals.

It is important to note that piezocapacitive sensors are highly sensitive to signal quality and sensor placement. Accurate biological signal acquisition necessitates complete contact with the skin and minimization of external interference. Additionally, as sensor size decreases, the measuring range of capacitive sensors can be limited due to the reduced surface area of the top and bottom electrodes, which may compromise sensitivity.^[^ ^95^ ^]^ Researchers have developed highly sensitive and miniaturized piezocapacitive sensors to address these challenges through materials engineering,^[^ ^96^ ^]^ mechanical design,^[^ ^97^ ^]^ and interfacial structure design advancements.^[^ ^98^ ^]^

##### Piezoelectric Sensing Mechanism

The piezoelectric effect is derived from the intrinsic properties of specific crystalline materials and specific polymers, as illustrated in Figure 3c. Notable examples include lead zirconium lead zirconium titanate (PZT),^[^ ^99^ ^]^ poly(vinylidene fluoride trifluoroethylene),^[^ ^100^ ^]^ and nylon.^[^ ^101^ ^]^ When subjected to mechanical deformation—such as compression, tension, or bending—these materials generate an electrical charge proportional to the degree of deformation. This phenomenon occurs due to the displacement of charge centers within the crystalline structure, resulting in a measurable voltage across the material.^[^ ^102^ ^]^

Like other elastic materials, piezoelectric substances can deform under applied stress and revert to their original shape once the stress is removed, provided the applied stress does not exceed their elastic limit. This behavior is consistent with Hooke's law, which governs mechanical effects:

(11) η=c×e

where *η* is stress, *e* is strain, and *c* is the elastic modulus, representing the force required for an object to produce a unit strain.

The resulting charge by piezoelectric effect can be drawn through the electrode to form a voltage signal as follows:

(12) V=−d·ΔP

where *V* is the output voltage (in *volts*), *d* is the piezoelectric charge coefficient (in *C/N* or *m/V*), and *ΔP* is the change in pressure (in *pascals* or *N/m^2^*).

The charge generated then can be calculated as follows:

(13) $$Q=d\cdot F=d\cdot \left(A\cdot \Delta P\right)$$

where *Q* donates the electric charge generated (in coulombs), *F* donates the force applied on the sensor (in newton*s*), and *A* donates the area of the sensor (in m^2^).

The versatility of piezoelectric sensors is attributed to their mechanical flexibility, extended lifespan, and chemical stability, rendering them suitable for deployment in various environments.^[^ ^103^ ^]^ Additionally, these sensors are classified as active devices, capable of converting mechanical inputs into voltage signals without requiring external power sources, distinguishing them from capacitive and piezoresistive sensors.

Despite these advantages, piezoelectric sensors encounter challenges, including sensitivity limitations and the necessity for meticulous material selection and preparation.^[^ ^104^ ^]^ Ongoing advancements in materials science and engineering aim to enhance the performance and applicability of these sensors, fostering innovation in smart technology and real‐time monitoring systems. As industries increasingly demand efficient and reliable sensing solutions, piezoelectric sensors continue to play a pivotal role in the evolving landscape of sensor technology.

##### Triboelectric Sensing Mechanism

The triboelectric sensing mechanism is based on the triboelectric effect of generating electrical charges when two dissimilar materials come into contact and are subsequently separated, as illustrated in Figure 3d. Typically, two insulating materials exhibiting a significant difference in their position within the triboelectric series are selected.^[^ ^105^ ^]^ When mechanical force is applied to these materials, they come into contact, resulting in the transfer of electrons due to variations in their electron affinity.^[^ ^106^ ^]^

The nanoscale surface roughness facilitates this charge transfer, and the charges remain localized on the surfaces upon separation, creating an electric field between them. This charge distribution forms an interface dipole layer, often called the triboelectric potential layer, which functions similarly to a capacitor, generating a potential difference that can be harnessed. The dipole layer remains stable due to the insulating properties of the polymers, preventing rapid dissipation of the induced charges.

As external mechanical forces compress the polymer layers, the distance between the charged surfaces decreases, increasing capacitance. Consequently, as the interplanar distance diminishes, the capacitance increases, thereby altering the generated voltage, as expressed in the following equation:

(14) Q=C·V

where *Q* donates charge, *C* donates capacitance, and *V* donates voltage.

The stored charges can flow when connected to an external load, generating an electric current. The current *I* can be expressed as:

(15) $$I=C\frac{dV}{dt}+V\frac{dC}{dt}$$

This equation indicates that the changing voltage *V* and capacitance *C* influence the current *I* due to mechanical deformation.

Triboelectric sensors operate similarly to piezoelectric sensors, generating electrical signals when subjected to dynamic pressures and functioning without needing an external power source.^[^ ^107^ ^]^ However, triboelectric technology sensors have stringent requirements regarding the contact quality between the sensor and the skin, necessitating stable motion for optimal performance.^[^ ^108^ ^]^ Additionally, signal processing and noise suppression present significant challenges for this technology, demanding the implementation of suitable algorithms and methods to enhance sensor functionality.

##### Piezomagnetic Sensing Mechanism

Piezomagnetic sensing is predicated on the piezomagnetic effect, wherein specific magnetic materials exhibit changes in magnetization in response to applied mechanical stress.^[^ ^109^ ^]^ As illustrated in Figure 3e, when mechanical stress is exerted, the material undergoes elastic deformation, leading to alterations in the internal structure of its magnetic domains. This deformation realigns the magnetic domains within the material, resulting in a measurable net change in magnetization. For example, Hall effect devices can measure this change by detecting the potential difference generated perpendicular to the current direction and the magnetic field when a conductor carrying an electric current is placed within the magnetic field.^[^ ^110^, ^111^ ^]^ Hall voltage *V_H_* can be quantified using the following equation:

(16) $$V_{H}=K\times B\times I$$

where *K* is the proportional constant, *B* is the magnetic induction, and *I* is the current.

Magnetic sensors are characterized by their non‐contact measurement capabilities, facilitating easy installation and offering strong resistance to ambient light and motion interference. As a result, the signal remains stable even under dynamic conditions.^[^ ^112^, ^113^ ^]^ This non‐contact feature enhances its suitability for invasive sensing applications.^[^ ^114^, ^115^ ^]^ Iron‐based amorphous alloys stand out among various materials due to their exceptional soft magnetic properties and a broad spectrum of sensitive physical effects. These materials demonstrate remarkable sensitivity to micro‐stress levels of ≤ 1 kPa, making them ideal for fabricating highly responsive components.^[^ ^116^, ^117^ ^]^

Piezomagnetic sensors present promising applications in hemodynamic monitoring; however, several limitations impede their broader implementation in this domain. These sensors are relatively large and exhibit high sensitivity to temperature fluctuations.^[^ ^118^ ^]^ Furthermore, electromagnetic noise from adjacent electronic devices can adversely affect sensor performance and reliability. While piezomagnetic sensors possess significant potential for hemodynamic monitoring, it is essential to address these limitations to enhance their effectiveness and reliability in clinical applications.

#### 3.1.2 Optical Sensing Mechanism

In addition to mechanical sensing mechanisms, researchers have developed flexible bio‐integrated sensors that utilize optical sensing mechanisms for hemodynamic monitoring by measuring the absorption properties of blood at the skin surface, as illustrated in Figure 3f. When hemoglobin in the blood absorbs light, the intensity of the transmitted light diminishes, and this change correlates with the hemoglobin concentration and the pulse signal.^[^ ^119^ ^]^ During each heartbeat, blood flow causes periodic fluctuations in light absorption, which can be detected by photosensitive elements and converted into electrical signals that reflect pulse waveforms.

Optical mechanism‐based flexible sensors for pulse monitoring primarily consist of a light source and photosensors.^[^ ^120^ ^]^ Light‐emitting diodes (LEDs) are commonly used as light sources, with wavelengths typically selected in the visible range, such as red light (660 nm) or near‐infrared light (700–1100 nm), to ensure effective skin penetration and absorption by blood.^[^ ^121^, ^122^ ^]^ Photodiodes are widely employed as photosensors to capture the light signals reflected from the skin.^[^ ^123^ ^]^ The output current signals generated by variations in light intensity can be calculated as follows:

(17) $$I=I_{0}\;\times 10^{-\varepsilon cld}$$

where *I* is the light intensity obtained at the time of measurement, *I_0_* is the initial light intensity without absorption by the skin tissue, *ε* is the molar absorption coefficient of a substance, which is a parameter describing the absorption capacity of a substance to a specific wavelength of light. *c* is the concentration of a substance, *l* is the thickness of skin tissue, and *d* is the optical path length.

Optical sensors, particularly those based on PPG, have gained prominence in hemodynamic monitoring due to their numerous advantages. These sensors are distinguished by their capacity for seamless integration into commercial products, such as smartwatches and fitness trackers, thereby offering a non‐invasive method for monitoring vital physiological parameters. Furthermore, their design inherently mitigates the effects of electromagnetic interference, a significant advantage in environments where electronic devices are prevalent.^[^ ^124^, ^125^ ^]^

The core functionality of PPG devices lies in their ability to detect volumetric changes in blood circulation through the absorption and scattering of light. As blood volume fluctuates with each heartbeat, these devices capture these variations and translate them into measurable signals, facilitating the assessment of metrics such as HR and blood oxygen saturation.^[^ ^122^, ^126^ ^]^ This integration into consumer electronics has not only enhanced the accessibility of health monitoring but has also paved the way for advancements in smart healthcare solutions.

Despite these advantages, optical sensors have limitations. A primary challenge is their susceptibility to ambient light interference, which can introduce noise into the signal and compromise measurement accuracy. Moreover, the effectiveness of optical sensors is significantly influenced by the sensing location and the lighting conditions under which they operate.^[^ ^127^ ^]^ In practical applications, additional factors such as scattering and blood oxygen saturation must also be considered; thus, integrating appropriate signal processing algorithms is essential for effective analysis and signal extraction.

#### 3.1.3 Ultrasonic Sensing Mechanism

At the core of ultrasonic sensing is the transmission of ultrasound waves, typically ranging from 1 to 20 MHz, into biological tissues.^[^ ^128^ ^]^ A transducer converts electrical signals into ultrasonic waves and vice versa, often utilizing piezoelectric materials for this purpose, as illustrated in Figure 3g. When these waves encounter moving scatterers, such as red blood cells with a specific frequency *f_0_*, they produce an echo that returns to the sensor at a frequency *f_R_*.^[^ ^129^ ^]^ The Doppler effect arises when relative motion between the sound source and the target occurs, resulting in a frequency shift *f_D_*. This frequency shift provides valuable information about blood flow velocity, which can be calculated using the following formula:

(18) $$f_{D}=f_{R}-f_{0}=\frac{2f_{0}cos\theta }{c}\;$$

where *c* is the speed of sound, *V* is the flow velocity, and *θ*, known as the Doppler angle, is the angle between the axis of the ultrasound beam and the direction of flow, looking toward the transducer. Unlike other sensing mechanisms, skin‐interfaced ultrasonic sensors can measure hemodynamics in deep tissues.^[^ ^130^ ^]^ However, this technique presents certain limitations, including the necessity for precise calibration, which can be influenced by tissue composition and movement, and the potential for signal interference from bubbles or other structures that may scatter the ultrasound, thereby compromising measurement accuracy.^[^ ^131^ ^]^ Additionally, ultrasound penetration is inherently limited, which may restrict its application in assessing deep blood vessels.^[^ ^132^ ^]^

Different mechanisms are suited to specific applications depending on factors such as energy efficiency, sensitivity, environmental compatibility, and depth of signal penetration. To ensure effective deployment of flexible bio‐integrated electronics for hemodynamic monitoring, it is essential to understand each sensing mechanism's unique advantages and limitations. **Table** **2** summarizes the key features, advantages, disadvantages, and typical application scenarios of the seven sensing mechanisms discussed in this section. Through the reference information, more appropriate sensing mechanisms can be selected to adapt to various application scenarios.

**Table 2:** Comparison of different sensing mechanisms and their application scenarios. Reproduced from^[^ ^81^ ^]^ with permission of BoD– Books on Demand.

Sensing Mechanism	Advantages	Disadvantages	Application Scenarios
Piezoresistive	Ease of acquisition and utilization Strong resistance to electromagnetic and vibration interference	Requires an external power source Signal drift over time The precision and linearity may be affected by environment	Monitoring blood pressure or pulse in areas with strong electromagnetic activity, such as intensive care units
Piezocapacitive	High sensitivity Good dynamic range Simple structure for fabrication	Requires stable external circuitry Sensitive to signal quality and sensor placement	Applications where precise pressure measurement is needed
Piezoelectric	Self‐powered and high energy efficiency Extended lifespan and chemical stability Fast response time	Limited sensitivity to small deformations Potential degradation over extended use in harsh environments	Continuous hemodynamic monitoring in outpatient or at‐home settings
Triboelectric	Self‐powered and high energy conversion efficiency Lightweight	Affected by environmental humidity Limited durability in dynamic conditions Stringent requirements for noise suppression	Motion‐induced signal acquisition during physical activity or fitness monitoring
Piezomagnetic	Non‐contact sensing capability High magnetic sensitivity Strong resistance to ambient light and motion interference	Requires an external magnetic field Limited integration potential Sensitivity to temperature fluctuation	Environments requiring non‐contact or remote sensing
Optical	High accuracy and resolution Multiple parameters measurement and seamless integration Mitigates the effects of electromagnetic interference	Requires external light source Susceptible to ambient light interference	Non‐invasive monitoring of blood flow and oxygen saturation, particularly in wearable or skin‐integrated devices
Ultrasonic	Penetrate deep tissues Accurate measurement of internal physiological signals	Relatively high‐power consumption Limited flexibility of some transducer designs	Imaging‐based monitoring of cardiovascular functions and detecting abnormalities within internal body tissues

### 3.2 Materials and Mechanical Considerations

Bio‐integrated sensors must satisfy several critical requirements: demonstrate stable performance in complex environments, exhibit stretchability to accommodate various skin or tissue deformations, and ensure biosafety, which encompasses biocompatibility and biodegradability.^[^ ^133^ ^]^ This section will discuss these requirements in detail, focusing on materials and structural design considerations.

#### 3.2.1 Environmental Stability Design

**Figure 4:**
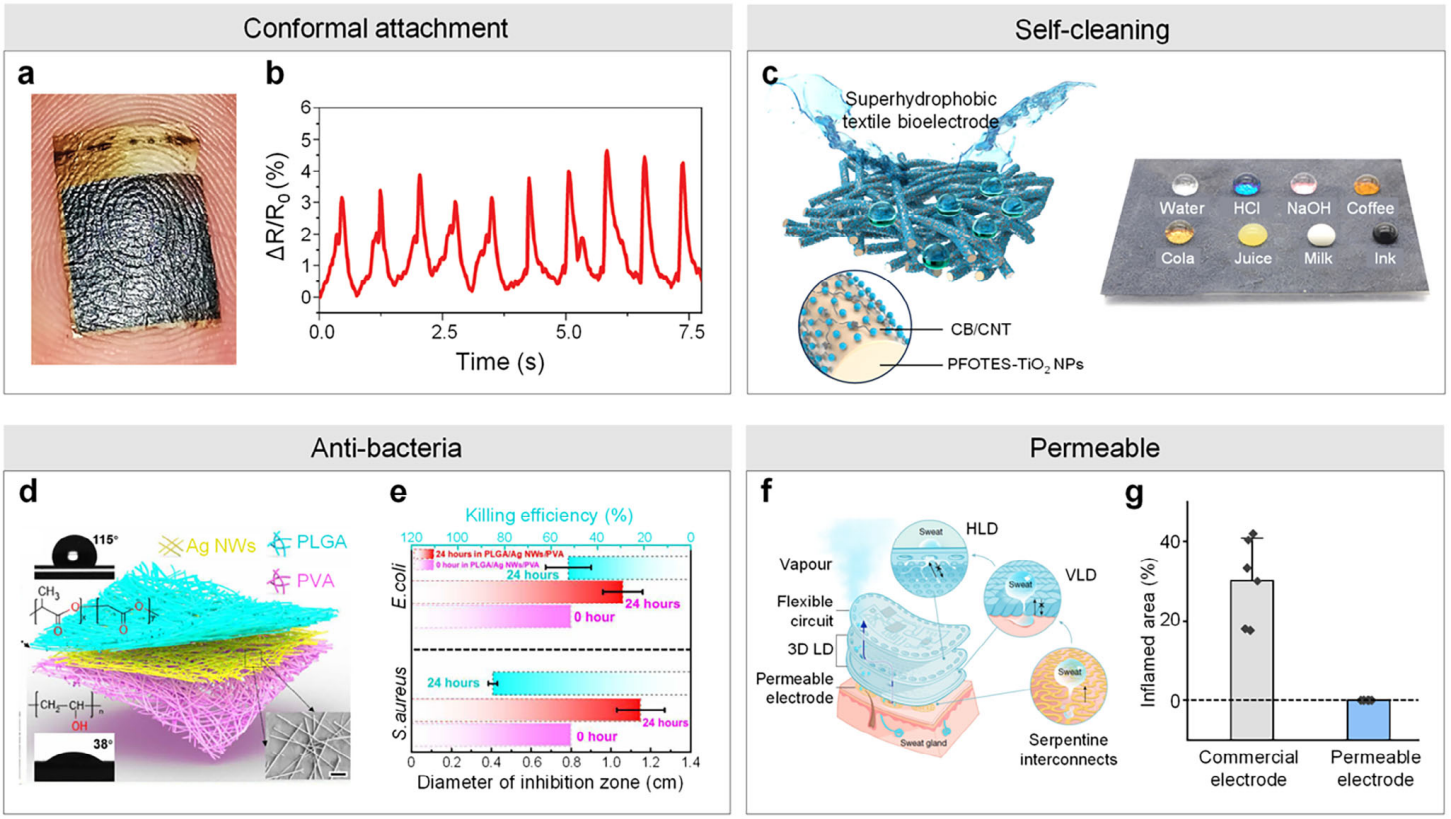
Materials and structures designed for high‐comfort bio‐integrated sensor development. a) Optical image presenting the LSG/PU nanomesh on the finger with clear fingerprint morphology. b) The detected pulse waves from the wrist artery. Reproduced with permission.^[^ ^136^ ^]^ Copyright 2022, Wiley‐VCH GmbH. c) Self‐cleaning bioelectronics based on superhydrophobic textile. Reproduced with permission.^[^ ^137^ ^]^ Copyright 2022, Elsevier. d) Schematic of the 3D network of the all‐nanofiber TENG–based E‐skin. e) Diameter of inhibition zone and antibacterial efficiency of the E‐skin. Reproduced with permission.^[^ ^138^ ^]^ Copyright 2020, American Association for the Advancement of Science. f) Illustration of the integrated system‐level sweat‐permeable electronics for CVD management. g) Skin‐irritation evaluation of different electrodes after long‐term wear. Reproduced with permission.^[^ ^140^ ^]^ Copyright 2024, Springer Nature.

Complex and variable skin interfacial environments significantly impact the sensing performance of bio‐integrated sensors and users' comfort. Engineers have developed ultrathin and conformal bio‐integrated sensors designed explicitly for hemodynamic monitoring to address this challenge.^[^ ^134^, ^135^ ^]^ The ultrathin nature of these sensors allows them to conform effectively to biological tissues with intricate surfaces, such as fingerprints. Inspired by this capability, Qiao et al. introduced a smart electric skin for hemodynamic monitoring incorporating laser‐scribed graphene and polyurethane (PU) nanomesh,^[^ ^136^ ^]^ as depicted in **Figure** **4**a. This innovative smart electric skin exhibits impressive sensitivity (GF ≈ 40) across a high linearity range (60%) and exceptional stability (> 1000 cycles). When applied to the wrist skin with water assistance, the smart electric skin can accurately monitor pulse waves in detail (Figure 4b).

Inspired by the self‐cleaning surfaces found in nature, various engineered surfaces have been developed that achieve comparable performance. Environments laden with dust and other contaminants can significantly impact the sensing capabilities of bio‐integrated sensors. Equipping these sensors with self‐cleaning surfaces presents an effective solution to this challenge. In this context, researchers have developed a range of self‐cleaning bio‐integrated sensors. For example, Dong et al. created a cost‐effective, nonwoven textile‐based skin‐integrated bioelectronic system for hemodynamic monitoring, which exhibits properties such as a skin‐like Young's modulus, ultra‐high stretchability, superhydrophobicity, and exceptional strain sensing and bioelectric detection performance,^[^ ^137^ ^]^ as illustrated in Figure 4c. The fabrication process involved evenly anchoring carbon black/carbon nanotube (CB/CNT) hybrids onto electrospun styrene‐ethylene‐butylene‐styrene (SEBS) nonwoven textiles, followed by uniform spraying of PFOTES‐TiO_2_ nanoparticles on the conducting layer. The resulting micro‐topological structure and low surface energy provided by the PFOTES‐TiO_2_ layer imparted water repellency and remarkable self‐cleaning capabilities to the textile‐based bioelectronic system. Furthermore, this system achieved an impressive gauge factor of up to 1134.7 within an ultra‐broad detection range of 1050.0%. It effectively captures pulse waves with detailed features when applied to the wrist.

Biological contamination poses a significant challenge for flexible bio‐integrated electronics. To address this issue, researchers have introduced antibacterial materials into these devices. For instance, Peng et al. developed a biodegradable and antibacterial electronic skin (E‐skin) based on triboelectric nanogenerators, which features silver nanowires (Ag NW) sandwiched between layers of polylactic‐co‐glycolic acid (PLGA) and polyvinyl alcohol (PVA),^[^ ^138^ ^]^ as depicted in Figure 4d. This all‐nanofiber structural design provides the E‐skin with a high specific surface area for contact electrification and numerous capillary channels that facilitate thermal and moisture transfer.

Additionally, the E‐skin demonstrated significant antibacterial activity against Escherichia coli and Staphylococcus aureus, effectively inhibiting bacterial growth and preventing infections (Figure 4e). Furthermore, this developed E‐skin achieved a remarkable sensitivity of 0.011 kPa⁻¹, enabling comprehensive monitoring of physiological signals, including pulse waves.

Additionally, flexible bioelectronics designed for long‐term application on the skin must maintain permeability to prevent skin inflammation. Engineers have developed various structured materials, including nanomesh‐based and porous flexible sensors,^[^ ^135^ ^]^ to achieve this functionality. For example, Zhang et al. presented a universal strategy utilizing a 3D liquid diode (3D LD) configuration for moisture‐permeable wearable electronics (Figure 4f).^[^ ^140^ ^]^ The 3D LD provides exceptional air and sweat permeability and facilitates directly integrating high‐performance flexible bio‐integrated electronics. Remarkably, even with prolonged wear, the fabricated device demonstrated reliable biosignal monitoring, exhibiting robust adhesion strength and ensuring comfort without irritation (Figure 4g).

#### 3.2.2 Stretchability Design

In addition to environmental stability, the self‐performance stability of flexible bioelectronics is crucial. Given that the skin experiences strains ranging from 0% to 80%,^[^ ^141^ ^]^ these devices must maintain stable sensing performance under various external mechanical stimuli. Bioelectronics are transferred from traditional silicon wafers to flexible substrates to achieve flexibility and conformability. Polyimide (PI) is commonly used in this process due to its high stability in complex environments.

**Figure 5:**
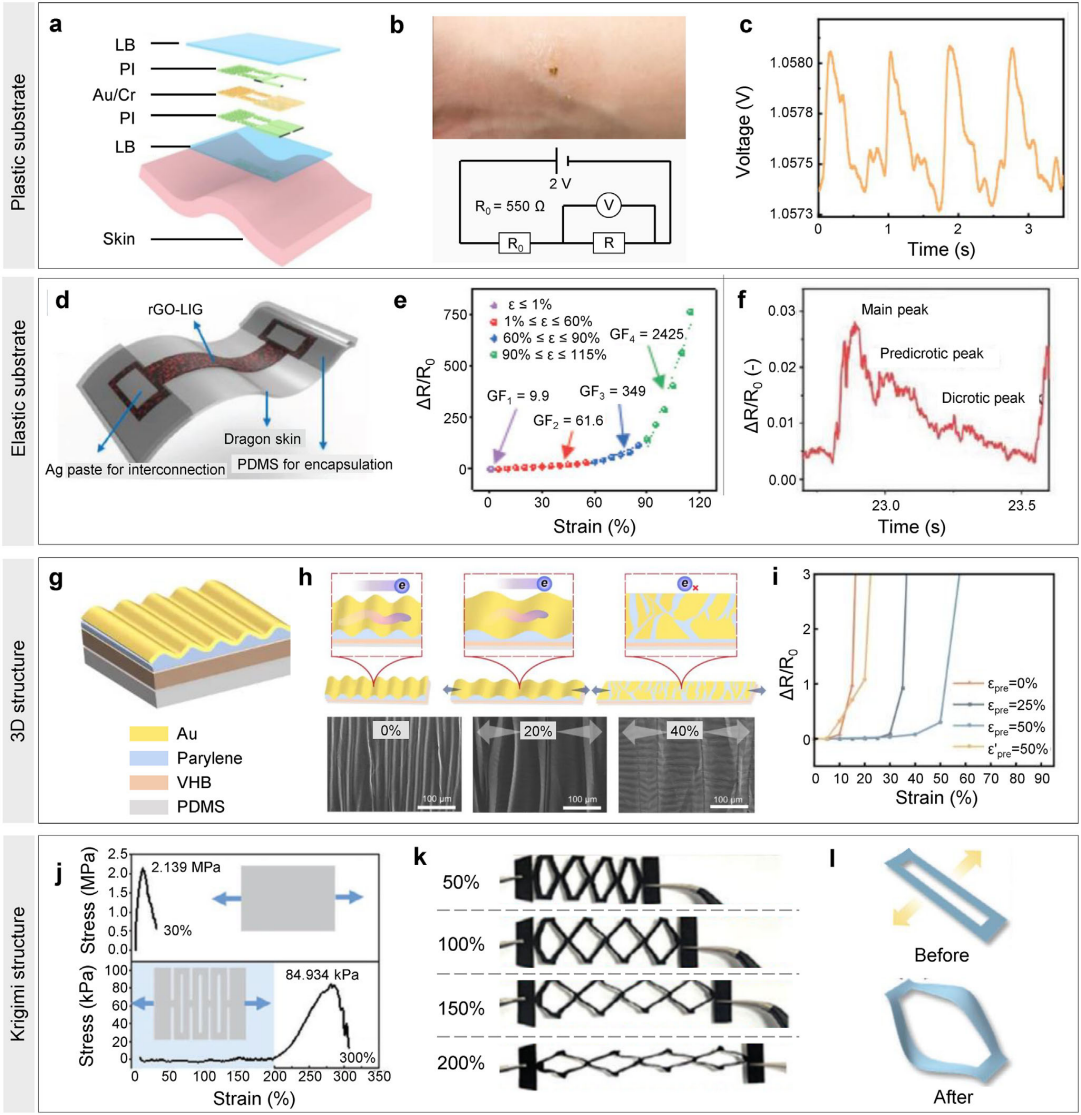
Materials and mechanical design for high‐stretchable pulse sensor development. a) Schematic of ultrathin strain sensor. b) Optical image of skin‐integrated ultrathin strain sensor for pulse monitoring. The insert below illustrates its electrical diagram. c) Collected pulse waves. Reproduced with permission.^[^ ^142^ ^]^ Copyright 2021, OAE Publishing Inc. d) Illustration of the FROS sensor based on Ecoflex substrate. e) Electromechanical performance and GF of the fabricated FROS strain sensor. f) Monitored pulse waveform with detailed features. Reproduced with permission.^[^ ^139^ ^]^ Copyright 2020, Wiley‐VCH GmbH. g) Illustration of wrinkle electrodes. h) Schematic and SEM images of wrinkled electrodes under different strains. i) Electromechanical response of different electrodes. Reproduced with permission.^[^ ^144^ ^]^ Copyright 2024, Wiley‐VCH GmbH. j) Stress‐strain curves for electrodes with different patterns. k) Optical images of fabricated devices under different tensile strains. l) Schematic of enhanced stretchability mechanisms induced by kirigami structures. Reproduced with permission.^[^ ^145^ ^]^ Copyright 2025, Wiley‐VCH.

For instance, Wong et al. detailed the development of an ultrathin skin‐integrated strain sensor that sandwiches an Au pattern between PI encapsulation layers (**Figure** **5**a).^[^ ^142^ ^]^ The patterned Au electrode, featuring a fractal curve, demonstrates a piezoresistive effect that is highly sensitive to ambient strain stimuli. Additionally, a liquid bandage layer was applied between the ultrathin strain sensor and the skin surface, serving as a connection layer to ensure intimate contact even on complex skin profiles. This close contact facilitates the reliable and precise monitoring of physiological signals while accommodating the user's unrestricted daily movements. The resulting strain sensor boasts an ultrathin profile of 6.2 µm and a weight of merely 0.204 mg. When positioned above the wrist artery on the skin surface, this ultrathin strain sensor effectively captures distinct pulse waves with intricate details, showcasing promising prospects for clinical applications (Figure 5b,c).

Although the polyimide (PI) substrate provides some flexibility, its stretchability is inherently limited to approximately 10%.^[^ ^12^ ^]^ Flexible sensors are integrated with more elastic polymers such as polydimethylsiloxane (PDMS), EcoFlex, SEBS, and polyurethane (PU) to enhance this stretchability. For instance, Yoon et al. developed a full‐range on‐body strain (FROS) sensor capable of accommodating both ultrasmall and large strain stimuli,^[^ ^143^ ^]^ as illustrated in Figure 5d. This device was fabricated by transferring reduced graphene oxide (rGO)‐embedded laser‐induced graphene (LIG) from a PI substrate to an EcoFlex substrate. The FROS sensor achieved an impressive gauge factor of 2445 across a strain range of 0–115% (Figure 5e). As anticipated, the FROS sensor effectively monitors physiological pulse signals with detailed waveforms (Figure 5f). Additionally, it can detect vocal sound waveforms and body movements and facilitate American Sign Language translation.

In addition to materials design, mechanical designs were widely utilized to extend the stretchability of soft bioelectronics. For instance, engineers have further developed various 3D structures to enhance stretchability.^[^ ^144^ ^]^ The prestretch‐release technique is a widely employed method for crafting 3D flexural structures, involving the application of high‐modulus inelastic conductive layers onto a pre‐stretched low‐modulus elastic substrate. Building on this concept, Chen et al. introduced a stretchable wrinkle polymer‐gold electrode^]^ depicted in Figure 5g.^[^ ^144^ ^]^ The fabrication involved depositing a thin parylene film on a pre‐stretched VHB/PDMS substrate and then depositing a gold layer. The higher Young's modulus of parylene (at the GPa level) compared to the pre‐stretched VHB (at the MPa level) led to the formation of a wavy folded structure in the PDMS/VHB/Parylene/Au composite, starting from the base upwards. During stretching, the amplitude of the fold patterns gradually decreased, eventually flattening out until reaching the initial pre‐stretching state (Figure 5h). As anticipated, the wrinkle electrodes, instead of those lacking pre‐stretch treatment, demonstrated an expanded strain range and maintained consistent relative resistance within their designated pre‐stretched strain thresholds (Figure 5i).

Furthermore, kirigami techniques have been harnessed to enhance stretchability in various applications. For instance, Shi et al. innovatively crafted a stretchable electrode by incorporating carbon black particles (CBs) onto paper with a meticulously designed kirigami pattern.^[^ ^145^ ^]^ Leveraging the kirigami structure, the engineered electrode demonstrated an impressive strain capacity of 300%, surpassing that of a conventional bulk electrode (Figure 5j). Despite this enhanced stretchability, the strength of the patterned electrode measured only 80 kPa, which was notably lower than that of a bulk electrode (2 MPa). The notable increase in stretchability can be primarily attributed to the out‐of‐plane deformation near the gaps within the kirigami structure, effectively redistributing stresses and maintaining structural integrity (Figure 5k,l).

#### 3.2.3 Biosafety Consideration

**Figure 6:**
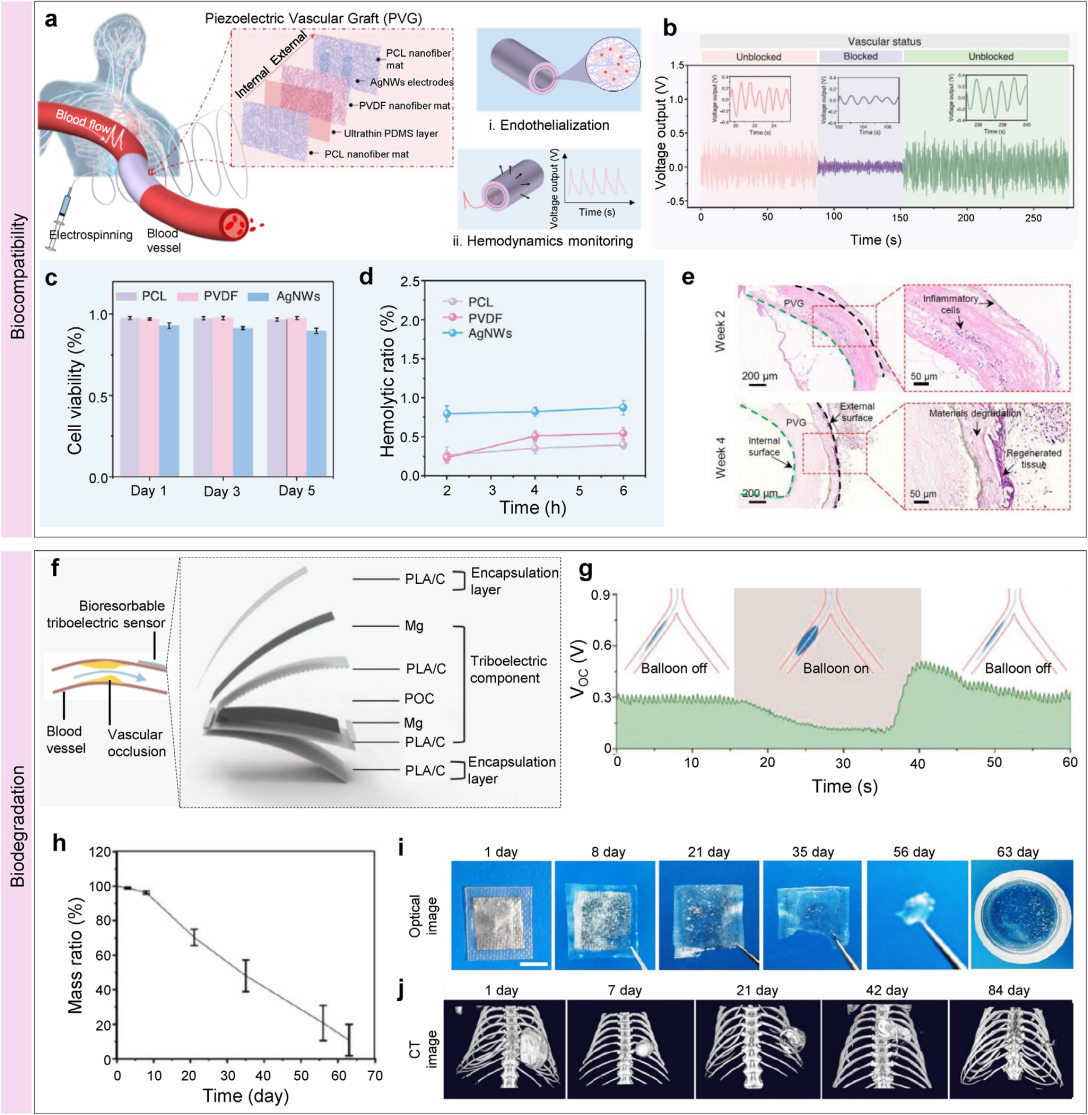
Biocompatibility and biodegradability of bio‐integrated sensors. a) Illustration of an all‐in‐one piezoelectric vascular graft for vascular healthcare. b) Detected hemodynamic information for vascular status evaluation. (c–(e) Cell‐, blood‐, and tissue‐level biocompatibility investigation results. Reproduced with permission.^[^ ^147^ ^]^ Copyright 2024, WILEY‐VCH GmbH. f) Structure of bioresorbable triboelectric pressure sensor. g) The implanted pressure sensor detects simulated abnormal vascular occlusion events. h) The mass ratio of the proposed pressure sensor responding to the degradation period. i) In vitro and j) in vivo degradation evaluation of fabricated pressure sensor. Reproduced with permission.^[^ ^148^ ^]^ Copyright 2021, WILEY‐VCH GmbH.

Since flexible bioelectronics are designed for long‐term interfacing with skin or soft tissues, the materials must be biocompatible to ensure biosafety.^[^ ^146^ ^]^ Commonly employed materials, such as polyimide (PI), polydimethylsiloxane (PDMS), EcoFlex, polycaprolactone (PCL), and polyurethane (PU), are well‐suited for the development of flexible bio‐integrated electronics. For instance, Ma et al. reported an all‐in‐one piezoelectric vascular graft (PVG) for hemodynamics real‐time monitoring and timely healthcare,^[^ ^147^ ^]^ as illustrated in **Figure** **6**a. The proposed PVG consisted of a polyvinylidene fluoride (PVDF) nanofiber mat with patterned silver nanowire (AgNW) electrodes and two PCL nanofiber mats functioning as package layers. The fabricated PVG device exhibited a high mechanical sensitivity of 11 mV/kPa, an assessment of hemodynamics with high accuracy. With collected hemodynamic information, the PVG could diagnose vascular status, for instance, vascular blockage (Figure 6b). Then, the biocompatibility of the fabricated PVG device was systematically investigated. Firstly, the cytocompatibility of the device was evaluated by using myofibroblast cells. Figure 6c shows no cytotoxicity exhibited by myofibroblast cells when cultured and sampled for up to 5 days. In addition, the hemocompatibility of the PVG device was evaluated. The experimental results revealed that the hemolytic ratio for all key materials used in PVG was less than 1%, illustrating remarkable hemocompatibility (Figure 6d). Moreover, tissue‐level biocompatibility was investigated through histological analysis, such as H&E staining, after embedding PVG devices into subcutaneous tissue for an extended period. As expected, the fabricated PVG device showcased excellent tissue compatibility, scaffold biodegradability, and tissue regenerative capabilities, as illustrated in Figure 6e. These findings confirmed that the fabricated PVG device showed remarkable biocompatibility and was suitable for long‐term usage.

For implanted devices, materials must be biocompatible and biodegradable. Biodegradable flexible electronics can be safely absorbed by the animal or human body after completing their intended function, eliminating the need for surgical removal. A variety of materials are suitable for the development of biodegradable electronics, including chitosan, silk fibroin, gelatin, polylactic acid (PLA), poly (lactic‐co‐glycolic acid) (PLGA), polyhydroxybutyrate valerate (PHB/V), and PCL. Ouyang et al. reported an implantable bioresorbable self‐powered sensor based on the triboelectric effect for vascular healthcare,^[^ ^148^ ^]^ as shown in Figure 6f. This device utilizes a triboelectric layer composed of poly (lactic acid) and chitosan (4%) (PLA/C) film with a nanostructured surface. In contrast, a magnesium (Mg) layer deposited on the backside is an electrode. Another PLA/C film, coated with Mg, is employed to create a nanostructured metal layer that functions as both an electrode and a triboelectric layer. The entire assembly is encapsulated in PLA/C to prevent liquid intrusion into the internal components of the sensor. When external force is applied, the two triboelectric layers come into contact, transferring electrons from the Mg surface to the PLA/C surface due to contact electrification, thereby altering the distance between the layers. The developed sensor demonstrated a high sensitivity of 11 mV/mmHg with excellent linearity (R^2^ = 99.3%). Based on collected hemodynamic information, animal tests revealed that the implanted pressure sensor could identify abnormal events, including vascular occlusions (Figure 6g). In addition to excellent mechanical sensing performance, biodegradation capability is another major performance of the proposed pressure sensor. As illustrated in Figure 6h, the mass of the fabricated pressure sensors was 98.9 ± 0.42% compared to the original status after culturing 3 days in a constant temperature phosphate buffer saline (37 °C, 1 × PBS). In addition, in vitro (Figure 6i) and in vivo (Figure 6j) experiments confirmed that the developed pressure sensor was degraded entirely after 63–84 days. As shown in **Table** **3**, typical materials employed for soft device development are summarized.

**Table 3:** Summary of typical materials for developing implantable sensors. Reproduced with permission.^[^ ^133^ ^]^ Copyright 2021, Wiley‐VCH Verlag.

Materials	Biosafety	Roles in sensors	Advantages	Limitations
*Metals*				
Gold (Au)	Biocompatible	Electrodes; wires; circuits; strain sensing	Chemical stable	
Titanium (Ti)	Biocompatible	Electrodes; wires; circuits; strain sensing	Adhesion layer	
Copper (Cu)	Biocompatible	Electrodes; wires; circuits; strain sensing	Easy fabrication; anti‐inflammatory properties	
Magnesium (Mg)	Biodegradable	Electrodes; wires; circuits	Easy processing; Essential nutrient	Fast biodegradation
Molybdenum (Mo)	Biodegradable	Electrodes; wires; circuits	Essential nutrient High strength & Youngs modulus	Slow biodegradation
*Silicon‐based materials*				
Silicon (Si)	Biocompatible	Substrate; strain sensing	Established microfabrication techniques	Rigid and stiff material
Silicon oxide (SiO_2_)	Biocompatible	Packaging layer; Dielectric layer	Established microfabrication techniques; Thermally stable;	
Silicon nitride (Si_3_N_4_)	Biocompatible	Packaging layer; Dielectric layer	Established microfabrication techniques; Thermally stable; High‐quality factor	
*Polymers*				
Poly (3,4‐ethylenedioxythiophene): poly (styrene sulfonate) (PEDOT:PSS)	Biodegradable	Electrode		Hydrophobic; Brittleness
Polyimide (PI)	Biocompatible	Substrate layer		
Polydimethyl siloxane (PDMS)	Biocompatible	Package layer		
Ecoflex	Biocompatible	Package layer		
Parylene C	Biocompatible	Substrate/Package layer		
Polyvinyl alcohol (PVA)	Biodegradable	Adhesive layer		
Polycaprolactone (PCL)	Biodegradable	Substrate layer		
Poly[octamethylene maleate (anhydride) citrate] (POMaC)	Biodegradable	Package layer		
Poly (glycerol sebacate) (PGS)	Biodegradable	Dielectric layer		
Polyhydroxybutyrate/polyhydroxyvalerate (PHB/PHV)	Biodegradable	Package layer		
Poly (1,8‐octanediol‐co‐citrate) (POC)	Biodegradable	Package layer		

## 4 Invasive Bio‐Integrated Sensors For Hemodynamic Monitoring

Invasive bio‐integrated sensors significantly enhance accuracy and effectiveness in detecting pathological changes and assessing disease progression by establishing direct contact with targeted internal biological tissues.^[^ ^149^ ^]^ These flexible sensors are typically integrated with the tissues through clinical procedures such as suturing or catheter delivery. This invasive monitoring approach provides clinicians valuable information for managing patient care during and after surgical operations.

For hemodynamic monitoring, invasive bio‐integrated sensors are categorized into two types: heart‐integrated sensors, which facilitate real‐time monitoring of cardiac function, and blood vessel‐integrated sensors, which focus on monitoring blood flow and pressure. The specifics of these sensor types are discussed below.

### 4.1 Heart‐Integrated Flexible Sensors for Hemodynamics Monitoring

As previously noted, the heart serves as the central organ of hemodynamics. Heart‐integrated sensors can directly measure hemodynamic parameters, accurately assessing cardiac function and the status of major blood vessels.

Dual et al. reported a significant advancement in managing post‐cardiothoracic surgery patients by developing a soft, flexible sensor made with biocompatible materials.^[^ ^150^ ^]^ When implanted on the heart's surface, this sensor enables continuous monitoring of heart volume, addressing the complexities of hemodynamic and volume management after surgery. As shown in Figure 5a, strain sensors in internal systems can record continuous hemodynamic signals. In contrast, pressure sensors are permanently implanted for short‐term use in the pulmonary artery, and the impedance catheter is primarily utilized in the experimental setting only. The mean ± standard deviation of EDVs measured by echocardiography, impedance, and strain are shown in Figure 5b, respectively. The authors demonstrated that the sensor provides real‐time data for up to two days on a tensile machine, showing improved accuracy in heart volume measurement compared to traditional clinical gold standards, with an error of only 7.1 mL for the strain sensor versus impedance and 14.0 mL versus ultrasound. This innovation allows for the early identification of complications and facilitates timely treatment decisions, ultimately enhancing patient outcomes following surgery.

However, the implantation and removal of these invasive sensors are often associated with surgical risks. Additionally, the need for frequent battery replacements can necessitate multiple surgical procedures, significantly increasing patient morbidity, mortality rates, and healthcare costs. In recent years, substantial advancements have been achieved in heart‐integrated monitoring devices, driven by the dedicated efforts of both the medical and engineering communities.

The currently dominant lithium‐ion batteries available on the market have notable limitations, including a limited lifespan and the requirement for regular replacement and recycling.^[^ ^151^, ^152^ ^]^ The latest generation of implantable heart monitors that rely on lithium batteries typically offers a functional lifespan of only about three years.^[^ ^153^, ^154^ ^]^ This heavy reliance on conventional electrochemical cell power sources constrains the operational longevity of implantable sensors, thereby hindering the potential for real‐time continuous biomedical monitoring. Consequently, the demand for sustainable power solutions has driven the development of biomechanical energy harvesting technologies, further encouraging the exploration of autonomous systems.^[^ ^155^ ^]^

**Figure 7:**
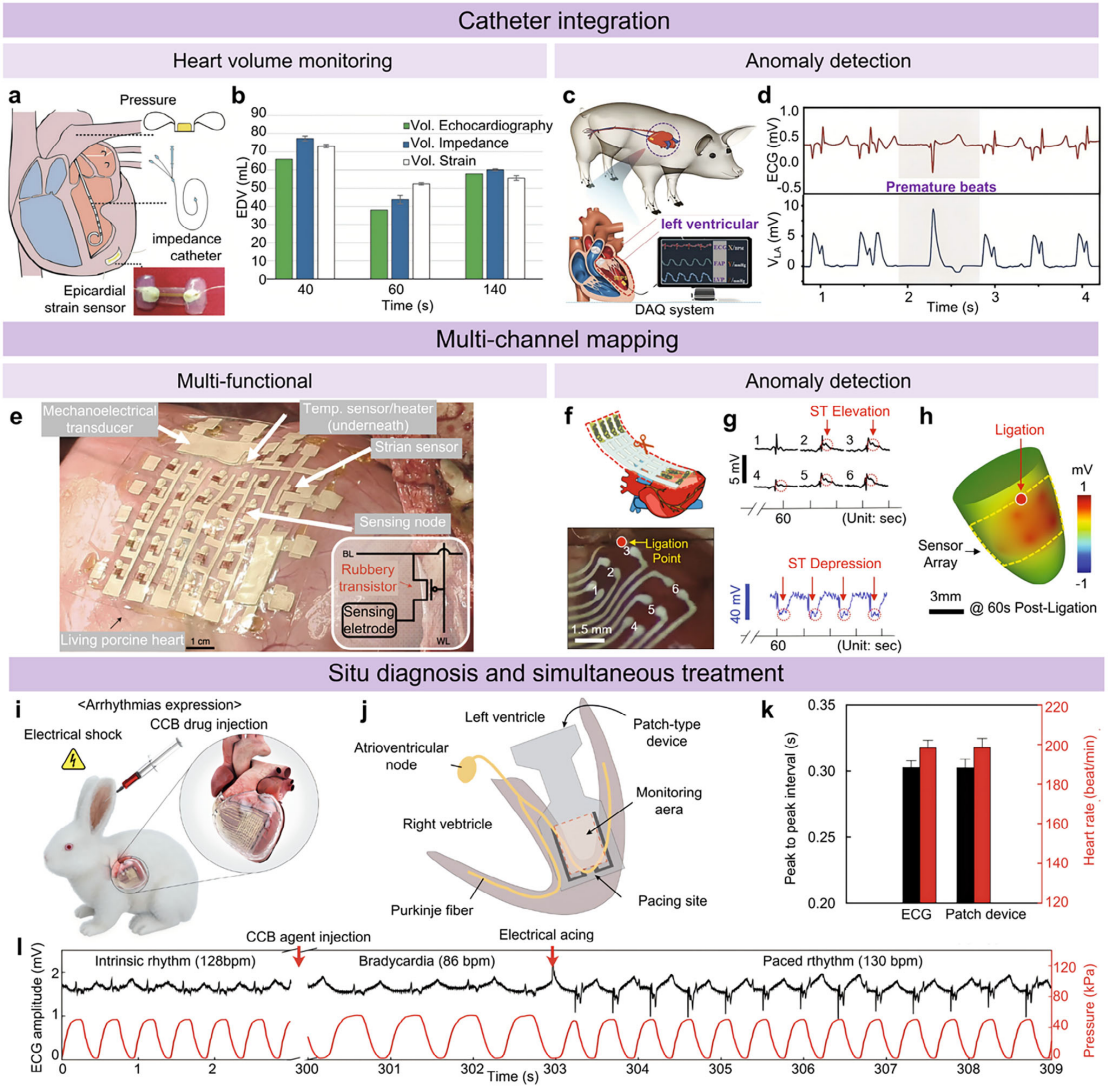
Representative heart‐integrated sensors. a) Internal systems record continuous hemodynamic signals. Pressure sensors are permanently implanted for short‐term use in the pulmonary artery, while the impedance catheter is mainly utilized in the experimental setting only. b) The mean ± standard deviation of EDVs at 40, 60, and 140 min after the start of the experiment was measured by echocardiography, impedance, and strain, respectively. Reproduced with permission.^[^ ^150^ ^]^ Copyright 2020, Wiley‐VCH GmbH. c) Schematic diagram of the semaphore acquisition from the SEPS implanted into an Adult Yorkshire swine's heart. d) Ectopic R waves in representative ECG indicating ventricular premature contraction corresponded to the enhanced waveform of the device. Reproduced with permission.^[^ ^163^ ^]^ Copyright 2019, Wiley‐VCH GmbH. e) The rubbery patch on the epicardial surface of a living porcine heart. Inset: the circuit diagram for a single sensing node in the 5 × 5 active matrix. BL, bit line; WL, word line. Reproduced with permission.^[^ ^164^ ^]^ Copyright 2020, Springer Nature. f) Implementation on the epicardial surface of the murine heart (Top) and the position of the myocardial infarction (Bottom). g) Measured epicardial ECG signals using the custom‐printed sensor array (Top) and simultaneously measured ECG signals using a control 3‐lead electrode set (Bottom). h) Post‐processed 3D image reconstructed from the spatiotemporally recorded epicardial ECG and ultrasound signals after 60 s post‐ligation. Reproduced with permission.^[^ ^165^ ^]^ Copyright 2021, Springer Nature. i) Schematic illustration on a live rabbit model for the arrhythmia expression by CCB drug injection and electrical shock. j) Schematic illustration on the cross‐section of a heart, indicating respective sites for monitoring cardiac beating motion and electrical pacing. k) Comparison of heart rates and the peak‐to‐peak interval derived from the pressure sensing and the surface ECG. l) In situ diagnosis and simultaneous treatment of bradycardia using the device platform. Restoration to intrinsic heart rhythm after electric pacing (0.6 V/mm, 2.2 Hz, 1 ms) using the device platform was performed while continuously monitoring epicardial pressures (red line) and surface ECG (black line). Reproduced with permission.^[^ ^166^ ^]^ Copyright 2022, American Association for the Advancement of Science.

Heart‐integrated sensors utilizing piezoelectric nanogenerators (PENGs) and triboelectric nanogenerators (TENGs) effectively harness mechanical energy and convert it into electrical energy, enabling self‐powered operation across various systems.^[^ ^156^, ^157^, ^158^, ^159^ ^]^ Typically constructed from soft materials to accommodate the shape and movement of the heart, these sensors are designed to be compact for implantation within cardiac tissues.^[^ ^160^, ^161^, ^162^ ^]^ For specific clinical populations, such as patients with heart failure characterized by impaired cardiac function, endocardial pressure (EP) is a crucial parameter for assessing heart performance. Traditionally, EP is monitored through invasive and costly cardiac catheterization, which is unsuitable for long‐term continuous data acquisition. Liu et al. reported a miniaturized, flexible, self‐powered endocardial pressure sensor (SEPS) based on a TENG that can be integrated with surgical catheters for minimally invasive implantation.^[^ ^163^ ^]^ In a porcine model, SEPS was successfully implanted in the left ventricle and left atrium, as illustrated in **Figure** **7**c. The sensor demonstrated effective responsiveness in both low‐ and high‐pressure environments, enabling ultra‐sensitive real‐time monitoring with a sensitivity of 1.195 mV/mmHg and exhibiting in vivo mechanical stability (R^2^ = 0.997). Additionally, SEPS could detect arrhythmias such as ventricular fibrillation and premature ventricular contractions, as illustrated in Figure 7d, with an excellent performance compared to gold standard devices ECG.

In addition to the catheter integration type, the flexible, stretchable sensor patch supports multi‐channel mapping and can be more widely wrapped around the surface of the heart. Sim et al. introduced a novel epicardial bioelectronic patch made from materials that match the mechanical softness of cardiac tissue.^[^ ^164^ ^]^ This innovative patch can perform spatiotemporal mapping of electrophysiological activity, sensing strain, and temperature, as illustrated in Figure 7e, demonstrating its functionality on a beating porcine heart. In addition to monitoring capabilities, the patch offers therapeutic functions, including electrical pacing and thermal ablation. This patch also integrates a rubbery mechanoelectrical transducer that can harness energy from heartbeats, potentially providing a power source for the device. The multifunctionality of the bioelectronic patch could assist physicians in cardiac monitoring and treatment decision‐making, ultimately aiding in discovering new therapeutic approaches. While primarily intended as a temporary epicardial implant, incorporating anti‐inflammatory agents and further investigating the effects of induced fibrosis may pave the way for the development of chronic implants. Kim et al. reported sponge‐like poroelastic silicone composites as the biocompatible inks adaptable for high‐precision direct writing of custom‐designed stretchable biosensors,^[^ ^165^ ^]^ which are soft and insensitive to strains, as shown in Figure 7f. The poroelastic nature of these devices allows for robust coupling to the epicardial surface, remaining insensitive to mechanical and electrical hysteresis associated with periodic cardiac and respiratory motion, further enhancing their reliability. In vivo, evaluations of epicardial ECG signals can be measured using the custom‐printed sensor array and control 3‐lead electrode set simultaneously, as shown in Figure 7g. The post‐processed 3D image in Figure 7h can verify that multi‐channel design can efficiently map the position of ST elevation. At the same time, the control 3‐lead electrode set just shows the event of ST depression. It demonstrates the clinical utility of the custom‐designed biosensors in a murine model of acute myocardial infarction, and the ability to simultaneously record epicardial ECG and perform real‐time ultrasound imaging showcases the dual functionality of these devices.

For clinical application, abnormal diagnosis implies the need for therapeutic intervention. The in situ diagnosis of cardiac activities with simultaneous therapeutic electrical stimulation of the heart is key to preventing cardiac arrhythmia. Hwang et al. proposed an unconventional single‐device platform for in‐situ monitoring and control of cardiac activity under humid conditions.^[^ ^166^ ^]^ To treat arrhythmias, the pacing electrodes integrated into the device platform can generate electrical impulses to control a rabbit's heart rate, and the pressure‐sensitive transistors continuously monitor its heart rhythm, as shown in Figure 7i. The alginate‐based hydrogel adhesive enables the sensor array to fit tightly onto the surface of the heart, ensuring that the device remains stable during heart movement and reducing signal interference. In this experiment, the device platform was placed on the anterior of the LV to monitor the rabbit's heartbeat, and the pacing electrodes were positioned near the apex to transmit the electrical pulses directly to the Purkinje fibers, as illustrated in Figure 7j. A good correlation was shown in Figure 7k between mechanophysiological readings and ECG, and there was little electrical interference when cardiac stimulation was performed. By injecting CCB, the rabbit's heart rate dropped from 128 to 86 beats per minute, and bradycardia became apparent, as depicted in Figure 7l. Electrophysiological and epicardial pressure tracks were observed on the ECG and the device platform, respectively. Electrical pulses (amplitude 0.6 V/mm; Pulse width 1 ms; Frequency 2.2 Hz) are used to pace the heart while continuously monitoring its heart movement to restore the rabbit's intrinsic heart rate after bradycardia. This platform can synchronize abnormal heart rhythms through effective cardiac pacing therapy without interfering with the recorded signals, demonstrating its potential application value in treating cardiac arrhythmias.

Heart‐integrated sensors are valuable tools for assessing critical parameters such as blood flow status, heart pump function, and myocardial contraction in patients with heart disease. However, these heart implants’ high production and surgical implantation costs pose significant challenges to their widespread adoption. Furthermore, the methods required for device maintenance and their limited service life present additional barriers to development. Moreover, any foreign object implanted within the human body can elicit an immune response, potentially triggering inflammation or rejection. These factors collectively constrain the applicability and popularity of this technology in laboratory settings, limiting its transition to clinical practice.

### 4.2 Artery‐Integrated Bioelectronics for Hemodynamics Monitoring

Blood vessel‐integrated sensors can directly assess various parameters, including BFV and BP. Such measurements can assist clinicians in making informed diagnostic and treatment decisions by delivering comprehensive insights into disease states, including vascular narrowing, thrombosis, and vascular dysfunction.^[^ ^167^, ^168^ ^]^ Motivated by this potential, researchers have developed a range of artery‐integrated sensors to enable real‐time hemodynamic monitoring.

**Figure 8:**
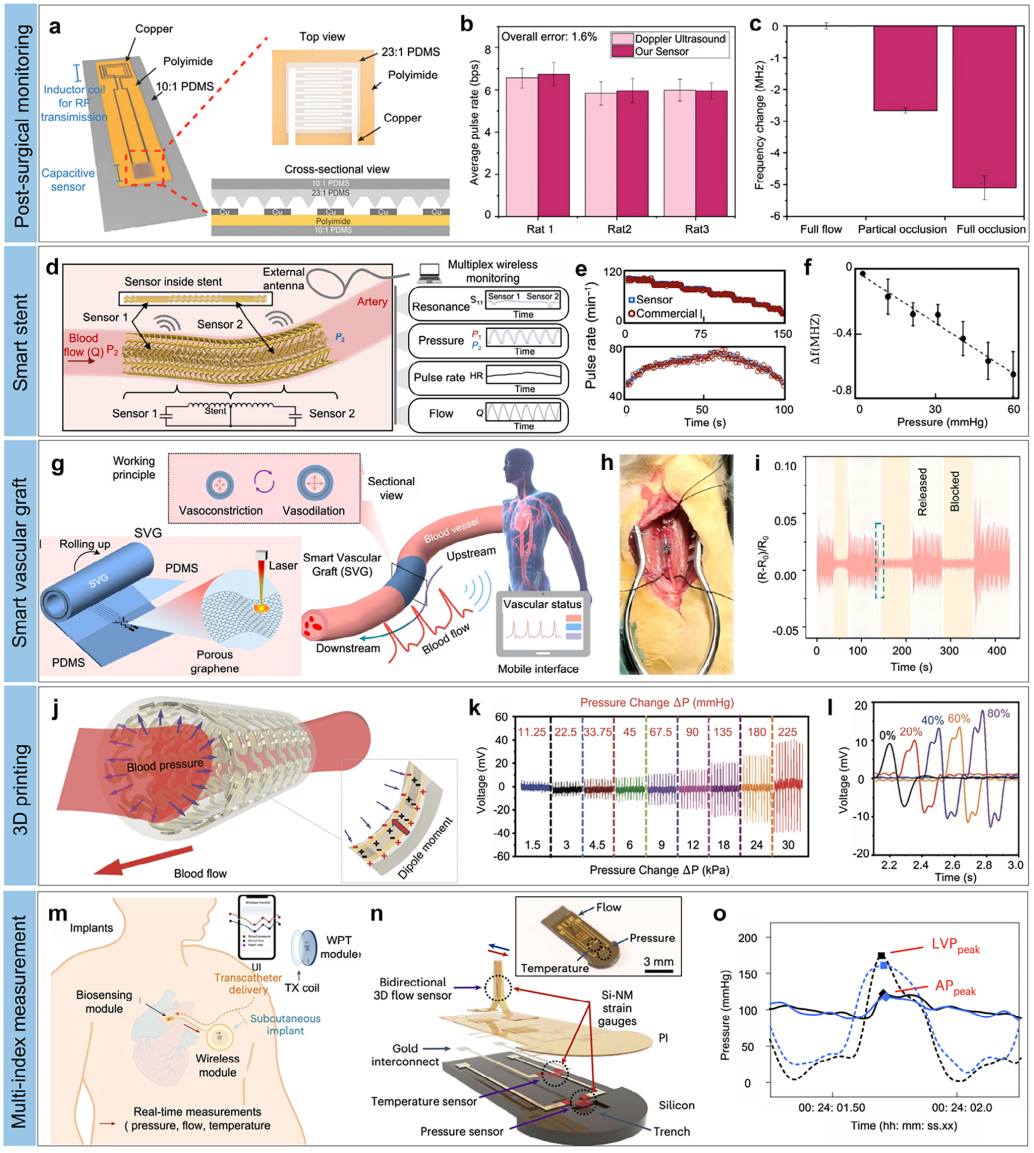
Typical artery‐integrated sensors. a) An exposed view illustration of the sensor with the inductor coil for wireless data transmission and interdigitated fringe field capacitive sensor to wrap around the artery. b) The pulse rate calculated from the Doppler ultrasound for each rat is compared to the pulse rate calculated from the wireless sensor readout. The overall error is 1.6%. Data are represented as mean change +/ SEM. c) The wireless sensor responds progressively to partial and complete occlusions 8.5 cm downstream of the sensor. Error bars indicate the range of the sensor response to pulsations for each condition, indicating signal variation due to arterial pulsations. Reproduced with permission.^[^ ^169^ ^]^ Copyright 2021, Elsevier. d) Illustration of the wireless design and sensing scheme to simultaneously monitor pressure, HR, and flow. e) Pulse rate detection during two flow conditions. f) Wireless pressure detection in an artery model. Reproduced with permission.^[^ ^170^ ^]^ Copyright 2022, American Association for the Advancement of Science. g) Illustration of the SVG system for hemodynamic monitoring and vascular healthcare. h) An optical image of the implanted SVG. i) Real‐time outputs of SVG experienced released and blocked vascular status. Reproduced with permission.^[^ ^171^ ^]^ Copyright 2025, American Chemical Society. j) Schematic illustration of the piezoelectric effect in the artificial artery in response to blood pressure. k) Voltage response of the artery as a function of pressure change. l) Comparison of detailed single voltage envelope under different levels of occlusion. Reproduced with permission.^[^ ^172^ ^]^ Copyright 2020, WILEY‐VCH GmbH. m) A battery‐free, implantable biosensing module that measures BP, BFV, and temperatures in the cardiovascular system, along with a wireless module, skin‐interfaced wearable module, and a WPT system. n) Schematic and exploded‐view illustration of the constituent layers: a laser‐cut silicon substrate, gold interconnects, temperature and pressure sensors mounted on flat and air‐filled substrate parts. o) Representative data of the measured pressure difference (ΔP = LVP−AP) across the AV compared with commercial devices. Reproduced with permission.^[^ ^76^ ^]^ Copyright 2023, Springer Nature.

For instance, Ruth et al. proposed a novel wireless capacitive sensor designed to be wrapped around the artery during surgical procedures, enabling long‐term monitoring of arterial health post‐operation.^[^ ^169^ ^]^ PDMS at a 10:1 PDMS to cross‐linker weight ratio is used as the encapsulation material, and the polyimide is used as the substrate, which has demonstrated strong biocompatibility in vivo. A lower‐modulus PDMS (23:1) is chosen as a pressure‐responsive element due to its biocompatibility, low compressive modulus for high‐pressure sensitivity, and negligible pressure response hysteresis, and the copper is selected as electrodes for electrical interconnect and wireless antenna, as demonstrated in **Figure** **8**a. This sensor is capable of continuously monitoring the health of the arteries, which is crucial for the early detection of complications such as arterial stenosis, decreased blood flow, and thrombosis, thereby helping to prevent severe cardiovascular events such as limb ischemia, strokes, and heart attacks. The overall error between the wireless sensor and the Doppler ultrasound was 1.6% in in vivo experiments, demonstrating a nearly identical response, as shown in Figure 8b. Notably, this sensor can respond to various artery sizes and degrees of occlusion, effectively monitoring changes at least 20 cm upstream and downstream of the sensor, demonstrating great flexibility and applicability. Figure 8c shows that the wireless sensor responds progressively to partial and complete occlusions 8.5 cm downstream of the sensor.

Moreover, experiments conducted on human cadavers and small animal models have shown that this sensor possesses strong capabilities for monitoring the progression of arterial occlusion, further validating its potential for use in actual clinical settings. The wireless monitoring capability of this technology provides new possibilities for the pre‐symptomatic detection and prevention of arterial health issues, allowing doctors and patients to understand the status of arterial health in real‐time, enhancing patient self‐management and improving treatment outcomes. This innovation has forward‐looking technological implications and holds significant preventive value in clinical practice.

Additionally, Herbert et al. reported a fully implantable vascular electronic system that integrates a wireless stent platform with printed soft sensors for real‐time monitoring of arterial pressure, pulse rate, and flow,^[^ ^170^ ^]^ as illustrated in Figure 8d. The stent structure utilizes conductive rings and non‐conductive connectors to establish a conductive path. At the same time, a flexible, low‐profile pressure sensor is laminated onto the inner surface of the stent, forming a pressure‐dependent inductance‐capacitance (LC) circuit. This system is monitored wirelessly through inductive coupling with an external loop antenna and a vector network analyzer (VNA) at communication distances significantly exceeding those of previous vascular sensors. The wireless electronic system was validated in artery models, and minimally invasive catheter implantation was demonstrated in an in vivo rabbit study. This innovative wireless system enables real‐time, simultaneous pulse rate, pressure and flow monitoring without batteries or complex circuits, yielding results comparable to a commercial pressure sensor. Figure 8e illustrates the pulse rate detection during two flow conditions, and Figure 8f indicates the implanted device's change in resonant frequency with low‐pressure ranges.

In addition to smart stents, researchers have developed smart vascular grafts for vascular healthcare. For example, Ma et al. reported a smart vascular graft (SVG) by seamlessly integrating vascular grafts with high‐sensitive flow biosensors,^[^ ^171^ ^]^ as shown in Figure 8g. The integrated flow biosensor was fabricated by encapsulating patterned porous laser‐induced graphene within biocompatible PDMS films. The working mechanism of SVG was described as follows: in the vasodilation process, there will be tensile strain acting on the SVG device, resulting in increasing electrical resistance; meanwhile, the vasoconstriction process recovered the SVG's dimensions to initial values, leading to a reduction in electrical resistance. The materials and mechanics of SVG were optimized, achieving remarkable electromechanical and mechanical performance. From its excellent mechanical performance, animal tests revealed that the fabricated SVG device could be implanted into the rabbit carotid artery (Figure 8h). The high‐sensitive porous graphene rendered SVG with outstanding mechanical sensing performance, featuring an ultralow strain detection limit of approximately 0.0034%. Based on the remarkable mechanical sensing performance, the implanted SVG could accurately detect hemodynamic information and further diagnose vascular status (Figure 8i).

3D printing technology has been introduced in artery‐integrated bioelectronics. For instance, Li et al. devised an electric field‐assisted 3D printing technique to create in situ‐poled ferroelectric artificial arteries, which provide battery‐free real‐time blood pressure sensing and occlusion monitoring.^[^ ^172^ ^]^ This innovative artery structure is enabled by creating a ferroelectric biocomposite that can be rapidly polarized during printing and reshaped into designed forms, as shown in Figure 8j. The exceptional piezoelectric performance is achieved through the synergistic interaction between potassium sodium niobate particles and a polyvinylidene fluoride polymer matrix. The high‐pressure sensitivity and capability to detect minute changes in vessel motion patterns facilitate early identification of partial occlusions, such as thrombosis, thereby helping to prevent graft failures. When the applied ∆P was boosted from 1.5 kPa (11.25 mmHg) to 30 kPa (225 mmHg), the voltage increased from 8.91 to 77.71 mV accordingly, depicted in Figure 8k, which evidenced that a complex 3D structure would not compromise the sensing accuracy. However, soft PDMS encapsulation could lower the output voltage at the exact pressure change. A comparison of a detailed single voltage envelope under different levels of occlusion (0–80%) of the artery system demonstrated that early thrombosis could be identified by the voltage profile, as shown in Figure 8l. This research showcases a promising approach to integrating multifunctionality into artificial biological systems for advanced smart healthcare applications.

Real‐time detection of multiple physiological parameters can significantly enhance the accuracy of health monitoring.^[^ ^12^ ^]^ Kwon et al. introduced a battery‐free wireless device that monitors various physiological indicators, including heart and blood vessel pressure, flow rate, and temperature,^[^ ^76^ ^]^ as illustrated in Figure 8m. The biosensing module employs strain gauges constructed from monocrystalline silicon nanomembranes (Si‐NMs) with a thickness of 200 nm, designed for measuring bi‐directional blood flow rate, pressure, and temperature around the heart. The multi‐layered structure consists of a laser‐cut silicon substrate, gold interconnects, and temperature and pressure sensors strategically mounted on flat and air‐filled regions of the substrate, respectively. A polyimide (PI) encapsulation layer with a thickness of 1.5 µm protects the device. At the same time, a bi‐directional flow sensor utilizes a 3D curvy ribbon created from a 2D precursor, as depicted in Figure 8n. This sensing patch is remotely controlled via a wirelessly charged Bluetooth low‐power system chip, enabling data presentation on an external user interface. Simultaneous measurements of left ventricular pressure (LVP) and aortic pressure (AP) using this wireless system in a sheep model demonstrated results comparable to those obtained from commercial devices, as shown in Figure 8o. The blood flow sensor exhibited high accuracy, with a negligible relative error of approximately 0.5 ± 0.4 L min^−1^, which aligns closely with the performance of commercial blood flow velocity sensors.

In addition, to eliminate the need for secondary invasive surgeries, bioresorbable blood vessel‐integrated sensors offer the potential to be absorbed by the body after their functional lifespan, representing a promising advancement in vascular healthcare.^[^ ^173^, ^174^, ^175^ ^]^ Artery‐integrated bioelectronics provide a transformative approach to hemodynamic monitoring by enabling real‐time, continuous assessments of blood flow and pressure through biocompatible, flexible, and multifarious‐form sensors, with the potential for multi‐parameter sensing, advanced data analytics, and significant clinical applications, while addressing challenges related to long‐term functionality, tissue compatibility, and cost‐effectiveness.

## 5 Non‐Invasive Bio‐Integrated Sensors for Hemodynamic Monitoring

Compared to invasive sensors, non‐invasive pulse sensors facilitate real‐time and continuous hemodynamic monitoring safely and comfortably. By maintaining direct contact with the skin, these sensors can convert subtle biomechanical signals generated by hemodynamics into electrical signals through various mechanisms, as discussed in Section 3.1.1.

**Figure 9:**
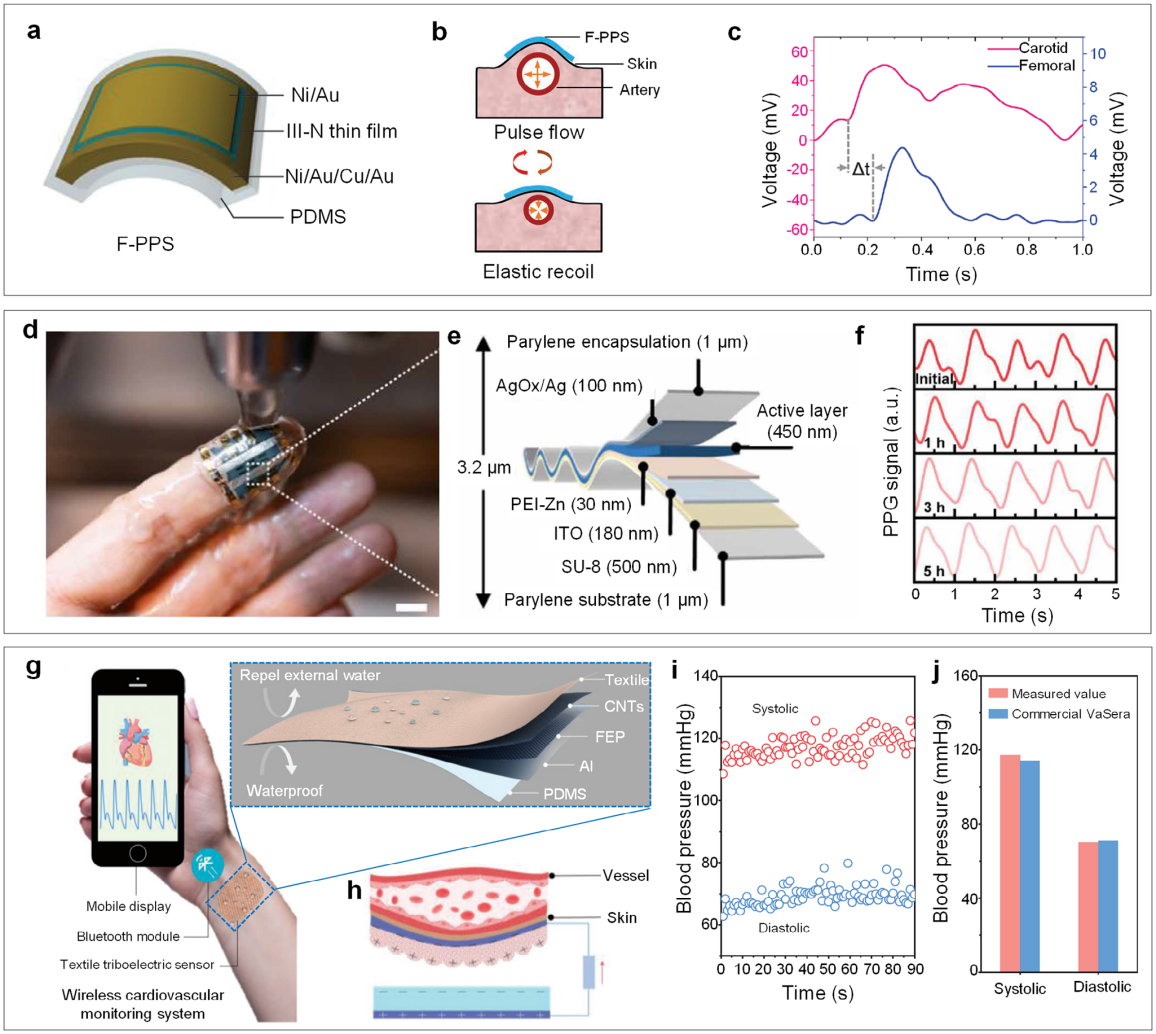
Ultrathin, high‐comfort pulse sensors for high‐accuracy monitoring of hemodynamics. a) Illustration of a thin, conformable piezoelectric pressure sensor. b) Working principle of the proposed pulse sensors. c) Pulse waves are detected from the carotid and femoral arteries. Reproduced with permission.^[^ ^177^ ^]^ Copyright 2018, WILEY‐VCH GmbH. d) Optical images of a fabricated OPD device. e) Schematic of the device structure of the ultrathin OPD. f) Collected pulse waves by skin‐integrated OPD for long‐term water immersion. Reproduced with permission.^[^ ^122^ ^]^ Copyright 2024, American Association for the Advancement of Science. g) Illustrations of designed cardiovascular system based on a self‐powered textile triboelectric sensor. The insert right showcases the structure of the textile triboelectric sensor. h) Schematic of the working principle of the skin‐integrated textile triboelectric sensor in response to the radial artery pulse. i) Estimated systolic and diastolic blood pressure using developed pulse sensors. j) Comparison of measured blood pressure with commercial cuff blood pressure gauge. Reproduced with permission.^[^ ^77^ ^]^ Copyright 2021, WILEY‐VCH GmbH.

Despite the advancements made in recent years, developing skin‐integrated pulse sensors that achieve high accuracy comparable to clinical standards, along with enhanced comfort and stability, remains a significant challenge that hampers their broader practical applications. Engineers have extensively addressed these challenges in materials engineering, structural optimization, and mechanical design. For example, Chen et al. developed an ultrathin and flexible piezoelectric pulse sensor (PPS) by employing a single‐crystalline group III‐nitride thin film,^[^ ^176^ ^]^ as illustrated in **Figure** **9**a. The proposed PPS device comprises an ultrathin III‐N thin film sandwiched by top Ni/Au electrodes and bottom Ni/Au/Cu/Au electrodes encapsulated by ultrathin PDMS films. The fabricated PPS device is ultrathin (≈300 µm in thickness), which could be attached to the skin surface conformally. As shown in Figure 9b, the working mechanism of the PPS devices for pulse wave detection could be described as follows: when the pulstical blood flow travels across the body, the expansion of the blood vessel will induce a subtle skin deformation above the artery; the pulse waves could bend the skin‐attached PPS device; the integrated piezoelectric sensing element could transfer this mechanical deformation into readable electric signal outputs. As discussed above, PWV is treated as an independent cardiovascular risk factor.^[^ ^177^ ^]^ PWV could be evaluated by collecting pulse waveforms at different arterial sites. As illustrated in Figure 9c, detailed pulse waveforms at the carotid and femoral arterial sites were collected. The PTT can be determined from the measured pulse waves as 98 ± 5 ms, and the corresponding carotid‐femoral PWV (cfPWV) can be calculated as 4.5 m s^−1^. The evaluated cfPWV values showcased a healthy cardiovascular status, in consideration of 10.0 m s^−1^ for cfPWV for diagnosis of arterial stiffness.^[^ ^178^ ^]^

The sensing performance of piezoelectric pressure sensors is significantly influenced by environmental factors such as movement, humidity, and temperature. In contrast, optical sensors demonstrate enhanced stability in complex environments, enabling the accurate measurement of hemodynamic parameters such as BP, HR, and PWV.^[^ ^42^, ^122^, ^179^ ^]^ However, many existing PPG sensors are rigid and thick, compromising user comfort. In addressing this challenge, Du et al. introduced an ultrathin and conformal organic photodetector (OPD) tailored for PPG signal detection, enabling reliable pulse waveform monitoring even in aquatic environments^[^ ^122^ ^]^ depicted in Figure 9d. At its core, this soft OPD comprised layers of indium tin oxide (ITO)/Zn2+‐chelated polyethylenimine (PEI‐Zn)/an active layer/AgOX/Ag, and a parylene encapsulation layer (Figure 9e). The active layer integration featured poly[4,8‐bis(5‐(2‐ethylhexyl)thiophen‐2‐yl)benzo[1,2‐b;4,5‐b′] dithiophene‐2,6‐diyl‐alt‐(4‐octyl‐3‐fluorothieno[3,4‐b]thiophene)‐2‐carboxylate‐2‐6‐diyl] (PBDTTT‐OFT) and poly[[N,N′‐bis(2‐octyldodecyl)‐napthalene‐1,4,5,8‐bis(dicarboximide)‐2,6‐diyl]‐alt‐5,50‐(2,20‐bithio‐phene)] (N2200) as the photoactive layer, with polystyrene‐block‐poly(ethylene‐ran‐butylene)‐block‐polystyrene (SEBS) elastomer serving as a filler. The OPD boasted an ultrathin profile of 3.2 µm, ensuring seamless integration and comfort on the skin surface. The OPD's robust linear photo response was noteworthy, and a mere 6% reduction in light current even after a 5‐h submersion in deionized water. This exceptional waterproof performance was likely attributed to the water‐resistant nature of the photoactive layer and mechanically reinforced interfaces surrounding it. As anticipated, this skin‐integrated water‐resistant device consistently delivered stable pulse waveforms with intricate details after 5 h of immersion, as illustrated in Figure 9f. However, the sensing performance of optical sensors is susceptible to ambient light interference and can be affected by variations in skin color,^[^ ^176^, ^177^, ^178^ ^]^ which may limit their applicability in specific contexts.

Furthermore, Fang et al. developed a wearable and conformal textile pulse sensor based on the triboelectric effect for long‐term and continuous pulse wave monitoring and cardiovascular,^[^ ^77^ ^]^ as illustrated in Figure 9g. The core element of the device was composed of fluorinated ethylene propylene (FEP) textile, a highly negative electron affinity material sandwiched by Al and single‐walled CNT electrodes. The device was packaged with an outer textile layer and an inner PDMS layer to make it waterproof. The fabricated pulse sensor was lightweight (0.27 g) and ultrathin (225 µm) and could be integrated on the skin surface conformally, achieving comfortable wearing for pulse wave monitoring. The proposed textile triboelectric pulse sensor could transfer biomechanical pressure induced by blood flow into electricity through triboelectric effect with electrostatic induction (Figure 9h). The pulsatile blood flow generated periodical pressure on the artery wall, producing mechanical deformation on the skin surface above the artery. The periodical mechanical deformation induced relative motion between CNT electrodes and FEP film, fulfilling the contact‐separation process and producing alternating currents. The fabricated pulse sensor exhibited remarkable electromechanical performance, including a high signal‐to‐noise ratio (SNR) of 23.3 dB, a short response time of 40 ms, and a high mechanical sensitivity of 0.21 µA/kPa, paving the way for pulse wave monitoring. The high‐performance pulse sensor could evaluate blood pressure by incorporating detailed pulse waves and custom‐developed algorithms. The estimated results revealed that the systolic and diastolic blood pressure were 117.3 and 70.2 mmHg, respectively (Figure 9i). Compared with clinical standards, the evaluated BP values showcased small mean deviations of only 2.9% and 1.2%, respectively (Figure 9j), validating high accuracy for BP evaluation.

**Figure 10:**
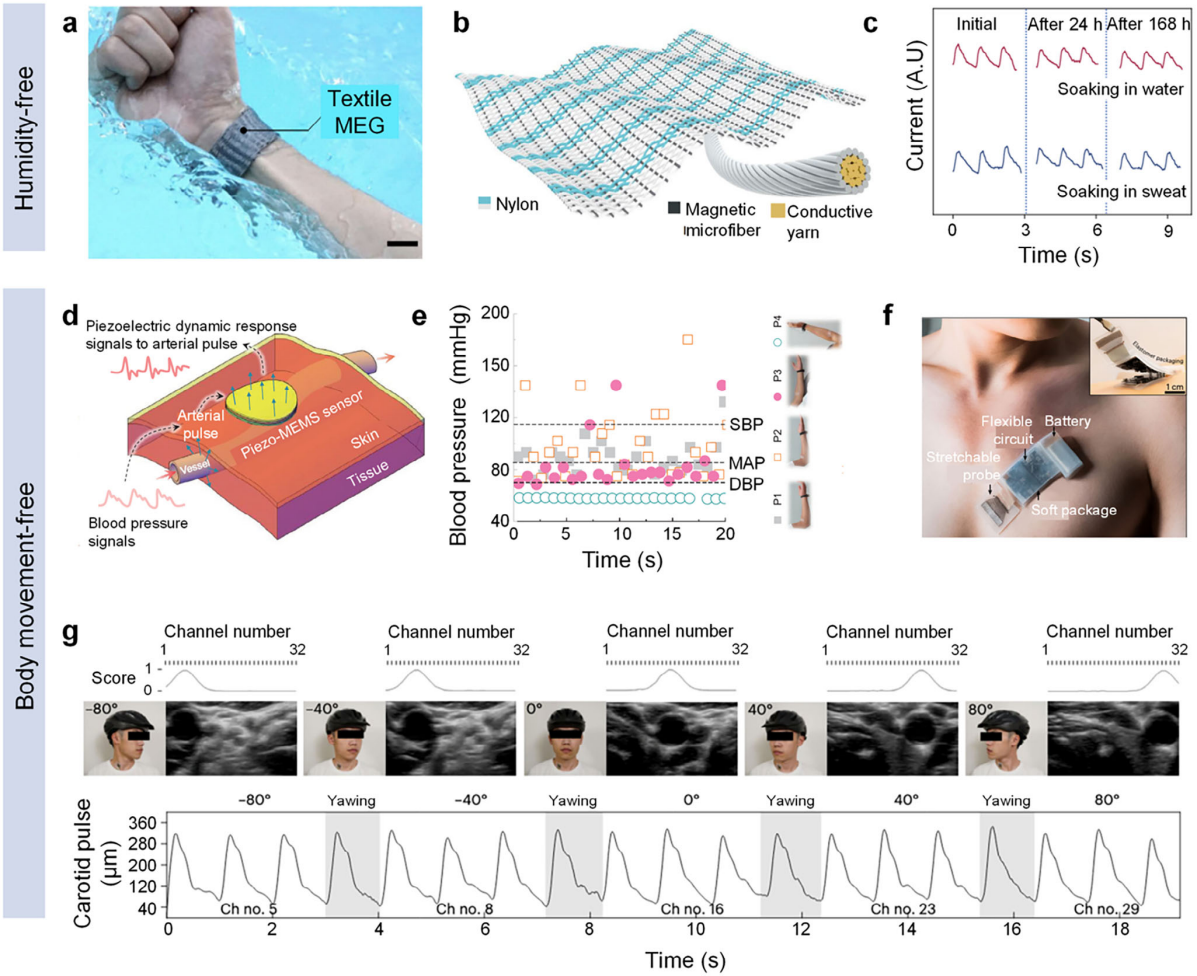
Skin‐integrated pulse sensors for stable monitoring of hemodynamics. a) Schematic of the designed textile MEG. b) The fabricated textile MEG was mixed with wool fibers to drive wearable electronics sustainably. c) The measured pulse waveforms were when the textile wristband was soaked in sweat and water for up to 168 h. Reproduced with permission.^[^ ^180^ ^]^ Copyright 2021, Springer Nature. d) Schematic of the designed wearable continuous BP monitoring system. e) Estimated BP at different postures. Reproduced with permission.^[^ ^181^ ^]^ Copyright 2022, Wiley‐Blackwell. f) Skin‐integrated ultrasonic system. g) BP evaluation results under different body postures. Reproduced with permission.^[^ ^182^ ^]^ Copyright 2024, Springer Nature.

In everyday life, the skin surface is subjected to complex and challenging conditions, including high humidity, significant skin deformation, and body movement. This necessitates the development of pulse sensors capable of stable hemodynamic monitoring under such variable conditions. Recently, Zhao et al. reported a highly stable textile pulse sensor based on the magnetoelastic effect,^[^ ^180^ ^]^ as shown in **Figure** **10**a,b. The developed soft fibers demonstrated superior magneto‐mechanical coupling compared to traditional magnetoelastic materials used in metal alloys. The soft magnetic fibers exhibit excellent humidity resistance, high current capabilities, and low internal resistance, enabling the textile pulse sensor to accurately detect high‐quality pulse waveforms, even in conditions of heavy perspiration or underwater scenarios, without additional encapsulation (Figure 10c). Furthermore, a fully standalone textile pulse sensor system was established by integrating wireless electronic modules and a customized mobile application, facilitating remote monitoring and telemedicine for multiple indicators, including HR, K value, PWV, stiff index (SI), and upstroke time (UT). In addition, Yi et al. demonstrated that motion artifacts caused by the variability in posture while acquiring the original arterial pulse wave could be effectively mitigated using the arterial pulse response (Figure 10d).^[^ ^181^ ^]^ They developed a wireless wearable continuous BP monitoring system based on a single piezoelectric sensor for pulse detection. This system achieved high accuracy and constant monitoring of BP, even under different arm postures, as illustrated in Figure 10e. The results validate that this BP monitoring system is more portable than those requiring multiple sensors to determine pulse wave speed. It highlights its potential for developing portable, wearable, continuous BP monitoring devices and for the early prevention and daily management of hypertension.

The non‐invasive techniques previously discussed are primarily limited to assessing hemodynamics within the superficial peripheral vascular system. While existing ultrasound technology enables non‐invasive observation of deeper tissues, challenges arise from the unstable coupling with the tissue surface, often exacerbated by conventional ultrasound probes' size and rigidity. In response to these limitations, Lin et al. introduced an autonomous wearable ultrasonic system fully integrated into a flexible patch. This system features a miniaturized control circuit that interfaces with an ultrasound transducer array for signal pre‐conditioning and wireless data communication,^[^ ^182^ ^]^ as illustrated in Figure 10f. To address movement artifacts and blood vessel movement caused by body movement, an auto‐selection algorithm was developed to select the best channel to follow the targeted carotid artery motion. To mitigate movement artifacts and account for the displacement of blood vessels due to body motion, an auto‐selection algorithm was developed to actively identify the optimal channel for tracking the targeted carotid artery. The results demonstrated that the wearable ultrasonic system could continuously monitor physiological signals from tissues as deep as 164 mm. Moreover, this ultrasound device is designed to conform to the skin's contours, capturing blood pressure waveforms from deeply embedded sites within arteries and veins, even at a thickness of 240 µm and a stretchability of up to 60% strain (Figure 10g).

## 6 Artificial Intelligence‐Assisted Signal Processing

**Figure 11:**
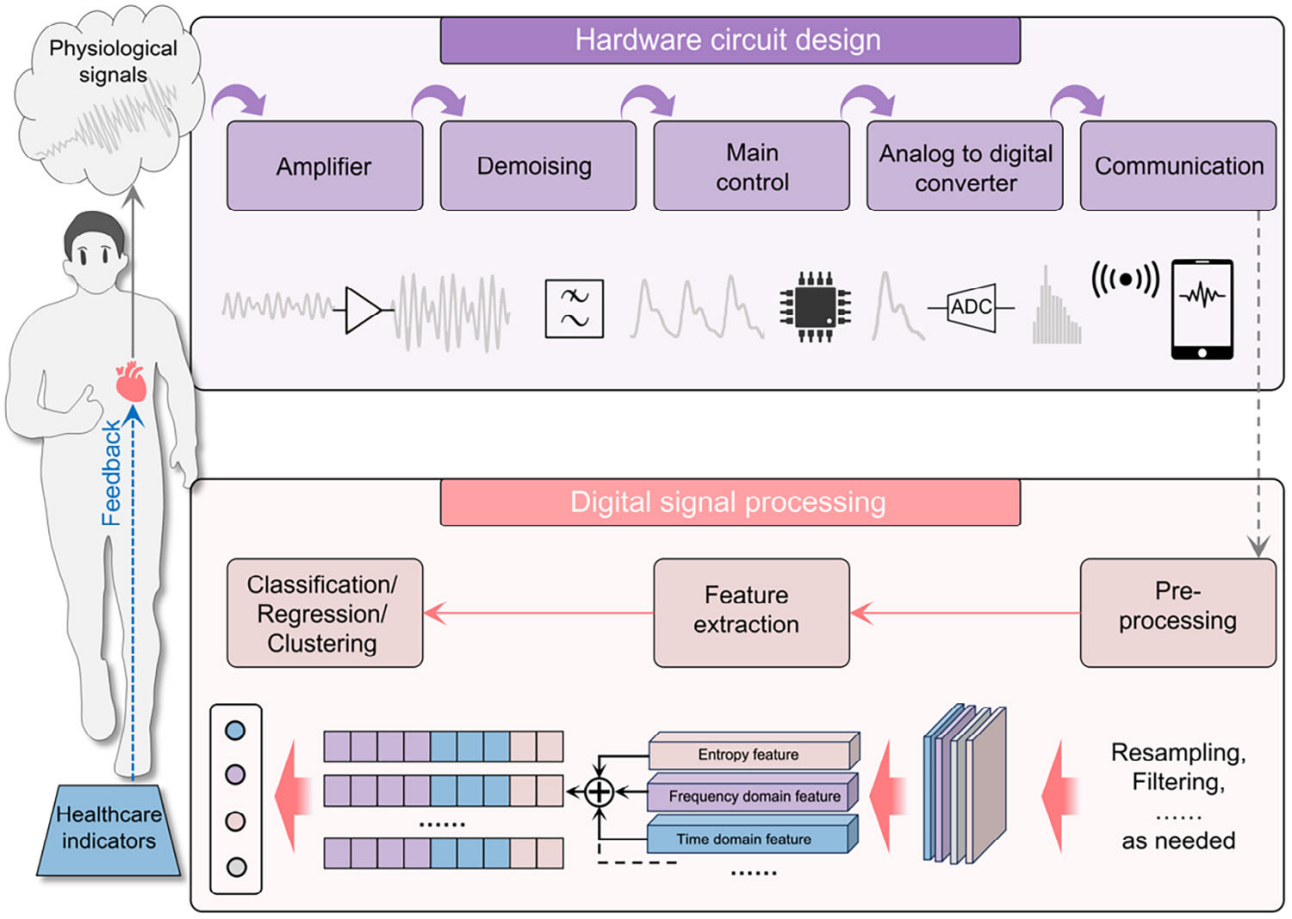
General signal processing flow.

Signal processing technology encompasses hardware and software components to establish a comprehensive sensing and analysis system, as depicted in **Figure** **11**. The hardware component provides the necessary electronics and circuitry for signal acquisition and conditioning, while the software component focuses on algorithmic processing and interpretation of the signals. This integrated approach enables developing advanced signal‐processing systems capable of extracting meaningful information from various physiological signals.

Typically, the raw pulse waveform signals obtained from sensing electrodes may contain noise and interference from multiple sources, and their amplitude can be relatively weak. Consequently, amplification and filtering circuits are required to enhance the signal quality.^[^ ^183^ ^]^ Once the analog signals are converted to digital format through analog‐to‐digital conversion (ADC), they undergo preprocessing to fulfill specific task requirements. Key features such as pulse rate, width, and amplitude can then be calculated.^[^ ^184^ ^]^ These features provide valuable insights into cardiac activity and can be utilized to monitor heart rate variability or detect irregularities in the pulse waveform.^[^ ^185^ ^]^

### 6.1 Data Pre‐Processing

The processes from signal input to the digital signal before feature extraction can be collectively called the data preprocessing stage. This stage encompasses hardware and software operations to optimize the digital signal for subsequent analysis. Various strategies can be employed to achieve high‐quality signals, including the application of amplifiers, careful signal conversion, selection of appropriate filters, and signal decomposition techniques.

Amplifiers play a crucial role in enhancing weak input signals, such as voltage, current, and power, transforming them into more robust outputs that facilitate effective signal collection and analysis. These devices are designed to achieve high input impedance and common‐mode rejection ratio while minimizing power consumption and size, particularly in biological applications.^[^ ^186^ ^]^ It can suppress interference signals to a certain extent, thereby improving the signal‐to‐noise ratio (SNR).

Analog signals, however, remain vulnerable to noise, interference, and attenuation. This vulnerability underscores the need for digital signal processing, which can effectively eliminate or reduce these issues, enhancing overall signal quality and accuracy while simplifying storage and transmission.^[^ ^187^ ^]^ Consequently, following the completion of hardware‐based signal processing, an analog‐to‐digital converter (ADC) is employed to transform the signal into a digital format for further operations.

Furthermore, the SNR can be enhanced through noise removal techniques implemented via analog or digital filters. Analog filters, designed using electronic components for processing continuous signals, are subject to performance limitations imposed by circuit elements and exhibit real‐time operation and bandwidth constraints. In contrast, digital filters are primarily utilized in digital signal processing,^[^ ^188^, ^189^ ^]^ enabling the application of complex algorithms and functions; however, they require sufficient computing power to facilitate real‐time feedback.

Various filter types, including low‐pass filters, adaptive filters, averaging smoothing filters, and Savitzky‐Golay smoothing filters, can be employed during the digital signal pre‐processing stage, tailored to the specific noise characteristics and the desired signal.^[^ ^190^, ^191^, ^192^ ^]^ For example, Jongshill et al. developed a third‐order Butterworth band‐pass filter to eliminate noise components, such as power line interference, baseline drift, and ambient noise, in wearable photoplethysmography systems utilizing multi‐channel sensors with multiple wavelengths.^[^ ^193^ ^]^ Typically, single‐stage filtering is insufficient to suppress complex pulse noise completely. In this context, Wang et al. analyzed the physical frequency components of pulse signals. They designed a cascade denoising approach that integrates a wavelet filter with a Gaussian filter, effectively mitigating white Gaussian noise and high‐frequency noise introduced by the analog‐to‐digital conversion process.^[^ ^194^ ^]^

In addition to filtering techniques, signal decomposition methods, such as wavelet transforms and empirical mode decomposition (EMD), can effectively isolate noise components from the underlying hemodynamic signals. Hossain et al. introduced an adaptive denoising method for ECG signals that combines ensemble empirical mode decomposition (EEMD) with thresholding techniques based on genetic algorithms (GA).^[^ ^195^ ^]^ This approach is superior to other denoising methodologies, as evidenced by improvements in signal‐to‐noise ratio, mean square error, and percent root mean square difference. Collectively, these advanced techniques facilitate the collection of high‐quality biosignals, providing a solid foundation for more accurate monitoring of hemodynamics.

### 6.2 Feature Extraction and Classification/Regression/Clustering

The significance of feature extraction is particularly pronounced in complex fields such as bioinformatics, where extensive data is generated from biological systems. In these contexts, feature extraction techniques are essential for reducing data dimensionality and identifying the most informative attributes for subsequent analysis. Once features are extracted, the next step involves training a model capable of classifying, clustering, or performing regression on new data based on these features. In hemodynamic monitoring of pulse waveform signals, various feature extraction techniques can be employed, which can be broadly categorized into two main groups: traditional machine learning (ML) algorithms that utilize predefined features and deep learning (DL) algorithms that automatically learn features from the data.

#### 6.2.1 Machine Learning Algorithms

Traditional features are crucial in capturing the underlying patterns and relationships within data, offering interpretable insights that enhance understanding of various aspects. The fundamental premise of ML is to uncover potential rules and correlations—referred to as features—through data learning and pattern recognition, which can then inform decision‐making processes. Typical traditional features can be categorized into several types: statistical features, spectral features, morphological features, waveform features, and wavelet‐based features, each applicable in time, frequency, and time‐frequency domains.

In the time domain, statistical features such as mean, variance, and maximum value can be computed alongside statistical morphological features like peak height and waveform width.^[^ ^196^, ^197^ ^]^ Frequency domain features encompass energy metrics such as band energy, total energy, and spectral morphology characteristics, including peak position and spectrum width.^[^ ^198^ ^]^ However, frequency domain features can be sensitive to noise and artifacts, often failing to capture the temporal dynamics or transient changes present in the signal.^[^ ^199^ ^]^

To address these limitations, time‐frequency domain features, such as those derived from wavelet transform and short‐time Fourier transform,^[^ ^200^, ^201^ ^]^ can effectively capture dynamic changes, frequency patterns, and irregularities, thus compensating for the deficiencies of single‐domain features.^[^ ^202^ ^]^

In contrast to linear features, which represent direct or proportional relationships, nonlinear features encompass complexities and dynamics, including multi‐scale sample entropy,^[^ ^203^ ^]^ Lyapunov exponent,^[^ ^204^ ^]^ Poincare graph, and independent component analysis.^[^ ^205^ ^]^ Nonlinear features often require computationally intensive algorithms, making their interpretation more complex than traditional linear features.

Moreover, rather than extracting features directly from the signal, model‐fitting features pertain to characteristics or parameters estimated by fitting a mathematical or statistical model to observed data. This process involves mathematical model parameters and prediction errors—such as those derived from Gaussian models and fitting residuals—which provide valuable insights into underlying patterns, trends, and relationships within the data.^[^ ^206^, ^207^ ^]^

Feature extraction methods can be selected and combined based on the characteristics of pulse waves and the specific analysis requirements, subsequently informing the choice of appropriate algorithms for training. Commonly employed algorithms are categorized into supervised, unsupervised, and semi‐supervised learning techniques.^[^ ^94^ ^]^

Supervised learning techniques are instrumental in scenarios where labeled data is available. For instance, the collected pulse wave signal cannot be directly translated into the corresponding BP value in BP prediction. Instead, known pulse wave signals are input into an ML model alongside their associated absolute BP values, which serve as labels for training. This process ultimately yields a predictive model that estimates BP values for new pulse wave signals.^[^ ^208^, ^209^, ^210^, ^211^ ^]^

**Figure 12:**
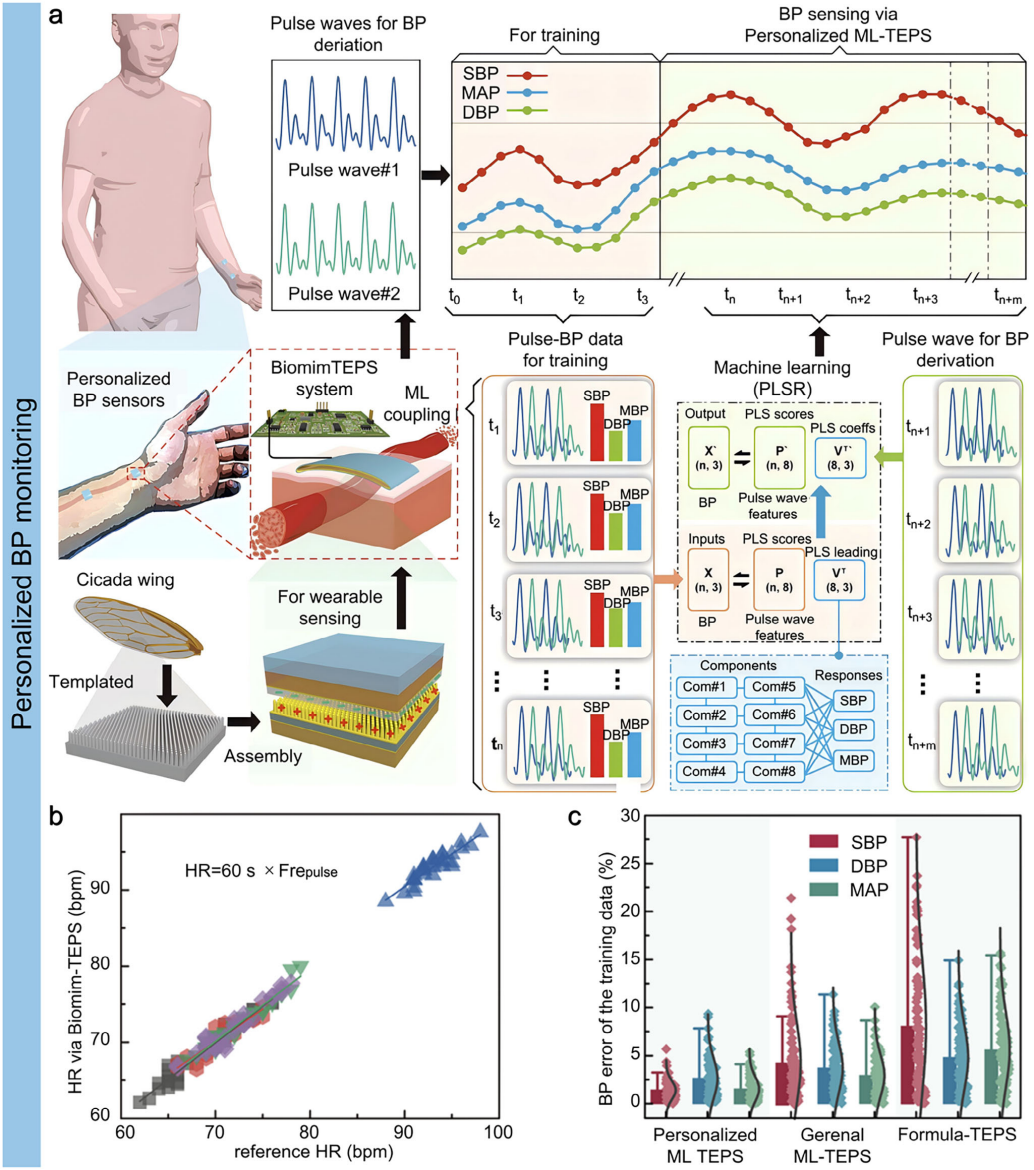
Personalized Machine Learning Pulse Sensor. a) Schematic showing the Biomim‐TEPS coupled with a Personalized Machine Learning algorithm to provide cuffless, accurate, and continuous BP monitoring. b) The scatter plot of the HR via the Biomim‐TEPS versus the reference HR measured by the cuff monitor. c) The statistical histograms showed the mean errors of the training group's SBP, DBP, and MAP derived via the Personalized ML‐TEPS, General ML‐TEPS, and Formula‐TEPS. Reproduced with permission.^[^ ^216^ ^]^ Copyright 2023, American Chemical Society.

These algorithms learn from the training data to generate predictions or classifications for previously unseen instances. They can be further divided into regression algorithms and classification algorithms. Regression algorithms, such as Linear Regression, Decision Trees (DT), Random Forests (RF), and Support Vector Regression, are employed for predicting continuous values. Conversely, classification algorithms, which predict discrete classes or categories, include Naive Bayes,^[^ ^212^ ^]^ DT, RF, Support Vector Machines (SVM),^[^ ^213^, ^214^ ^]^ and k‐nearest Neighbors.^[^ ^215^ ^]^ For instance, Yao et al. described a triboelectric pulse sensor Biomim‐TEPS that utilizes a biomimetic nanopillar layer.^[^ ^216^ ^]^ The cicada wing‐templated CNTs/PDMS nanopillars could effectively enhance the triboelectric performance, allowing sensitive detection of weak signals of rhythmic pulse waveform in a self‐powered manner. It is integrated with a personalized Partial Least‐Squares Regression (PLSR) algorithm, a typical linear regression model that provides cuffless, accurate, and continuous blood pressure monitoring, as demonstrated in **Figure** **12**a. Each participant's pulse wave signs and corresponding blood pressure data in the first two days will be collected in personalized monitoring. The partial least squares regression (PLSR) model will be used for training to generate a personalized sphygmo‐blood pressure relationship model, which is beneficial to overcome the inaccuracy of sensors with universal designs when applied to individuals. The pulse wave frequency measured via the Biomim‐TEPS compared to the reference HR measured by the cuff monitor is shown in Figure 12b. Most of the HR data points were distributed around the statistical plot's diagonal, indicating the accuracy of HR measurements via the Biomim‐TEPS. The deviation of all BP data derived by the Biomim‐TEPS from the reference BP in training groups was presented in Figure 12c. The training deviations based on Personalized ML‐TEPS were distributed with a lower average error of < 3%, while the General ML‐TEPS and Formula‐TEPS methods possessed larger training deviations in the range of 5% to 8%, confirming that the Personalized ML‐TEPS method can derive BP with higher accuracy.

**Figure 13:**
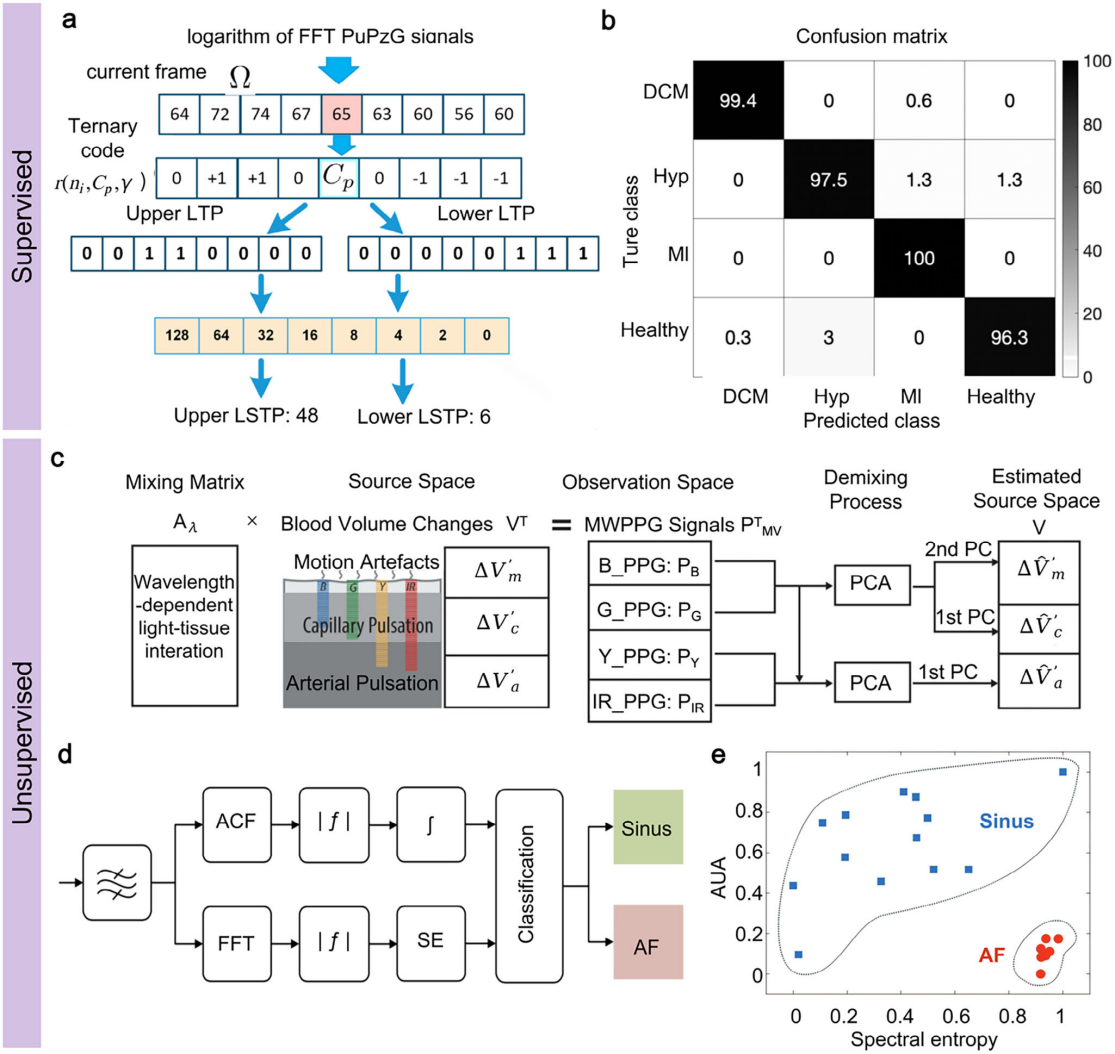
The typical ML methods for pulse waveforms. a) The procedure for computing the 1D‐LSTPs from the segmented PuPG signal frame. b) A class‐wise performance evaluation of the confusion matrix. Reproduced with permission.^[^ ^213^ ^]^ Copyright 2022, Wiley‐Blackwell. c) PCA‐based multi‐wavelength photoplethysmography algorithm. Reproduced with permission.^[^ ^223^ ^]^ Copyright 2021, Inderscience Publishers. d) Pipeline for the sinus rhythm and AF detection algorithm. e) The clustering of healthy and AF patients using time‐frequency analysis. Reproduced with permission.^[^ ^224^ ^]^ Copyright 2019, Nature Research.

In addition, SVM has gained significant traction in processing hemodynamic signals, achieving high accuracy in BP prediction.^[^ ^213^, ^217^ ^]^ For example, Sumair et al. developed a computer‐aided diagnosis system aimed at detecting Myocardial Infarction (MI), Dilated Cardiomyopathy (DC), and Hypertension (Hyp) from pulse plethysmograph (PuPG) signals.^[^ ^213^ ^]^ They preprocessed the raw PuPG signals using EMD and extracted highly discriminative features through a novel local spectral ternary pattern (LSTP) approach, as illustrated in **Figure** **13**a. A comparison between the proposed LSTP features and time and spectral domain features advocates the high discriminative power of LSTP features. The extracted LSTPs were input into various classification methods, including SVM, KNN, and DT. Notably, the SVM with a cubic kernel achieved the best classification performance, yielding an accuracy of 98.4%, sensitivity of 96.7%, and specificity of 99.6% through 10‐fold cross‐validation, as shown in Figure 13b.

However, not all datasets come with known labels for training. Unsupervised learning algorithms are employed in cases that do not rely on labeled data. Instead, these algorithms uncover patterns, relationships, and structures within the data without redefined labels or target variables. Popular unsupervised learning algorithms include hierarchical clustering,^[^ ^218^, ^219^ ^]^ Principal Component Analysis (PCA),^[^ ^220^ ^]^ K‐means clustering, and Association Rules mining.

To reduce the complexity of input signals, the application of Principal Component Analysis (PCA) can effectively eliminate redundant information and decrease data dimensionality by extracting the principal features that capture the majority of variance. Saleh et al. proposed two Pulse Shape Discrimination (PSD) techniques utilizing Cross Correlation (CC) and PCA.^[^ ^221^ ^]^ Liu et al. developed a multi‐wavelength PPG (MWPPG) strategy for blood pressure estimation using a single sensing node, introducing an enhanced algorithm to extract arterial pulsation, capillary pulsation, and motion artifacts based on PCA.^[^ ^222^, ^223^ ^]^ The MWPPG signals in the observation space can be derived from the source pulsation space through a wavelength‐dependent light‐tissue interaction process, represented by a mixing matrix, as illustrated in Figure 13c. The results indicate significant accuracy improvements enabled by PCA‐based operations on MWPPG signals, yielding errors of 1.44 ± 6.89 mmHg for SBP and −1.00 ± 6.71 mmHg for DBP.

These algorithms explore the inherent structures within data to identify clusters or associations without relying on labels, making them particularly effective for detecting physiological abnormalities. Kaisti et al. developed a flexible, wearable wristband utilizing a commercial microelectromechanical (MEMS) pressure sensor array to diagnose clinical cardiovascular events.^[^ ^224^ ^]^ They implemented a two‐line pipeline for atrial fibrillation (AF) detection, incorporating a band‐pass filter. The first line consists of autocorrelation, absolute value, and integration, while the second line employs fast Fourier transform, absolute value, and spectral entropy, as illustrated in Figure 13d. The features from both lines are then fused for final classification, and clustering analysis demonstrates high classification accuracy between AF and sinus rhythm, as shown in Figure 13e.

Semi‐supervised learning enhances model performance by leveraging both labeled and unlabeled data. This approach utilizes the distribution information and similarities in unlabeled data to assist in training models.^[^ ^225^ ^]^ The effective use of unlabeled data can significantly improve model performance, particularly when labeled data is scarce. By combining labeled and unlabeled data during the training process, the dataset's information is utilized more effectively, enhancing the model's generalization ability and accuracy.

#### 6.2.2 Deep Learning Algorithms

Through pulse waveform analysis, ML algorithms have demonstrated considerable potential in enhancing cardiovascular health monitoring and disease diagnosis accuracy and efficiency.^[^ ^226^ ^]^ However, effective feature selection still necessitates the application of domain knowledge.^[^ ^227^ ^]^ In contrast, DL facilitates the automatic extraction of useful features from multidimensional data, streamlining data preprocessing and enhancing recognition performance. By training DL models, both automatic feature extraction and classification are achieved, significantly improving the accuracy and efficiency of signal processing. This approach enables personalized health monitoring and early warning systems tailored to individual characteristics and historical data.

Convolutional Neural Networks (CNNs), primarily recognized for their applications in image analysis, can also be effectively employed for pulse waveform analysis.^[^ ^228^ ^]^ By treating pulse waveforms as 1D signals, CNNs can learn spatial features and patterns, which proves advantageous for various tasks, including detecting abnormalities, classifying different pulse waveform types, and predicting physiological parameters.

Recurrent Neural Networks (RNNs), including variants such as Long Short‐Term Memory (LSTM) and Gated Recurrent Unit (GRU), are particularly adept at analyzing temporal dependencies in pulse waveforms.^[^ ^229^ ^]^ These algorithms can identify patterns and trends over time, facilitating tasks such as predicting future pulse waveform values, detecting anomalies or irregularities, and monitoring changes in the waveform across extended periods.

**Figure 14:**
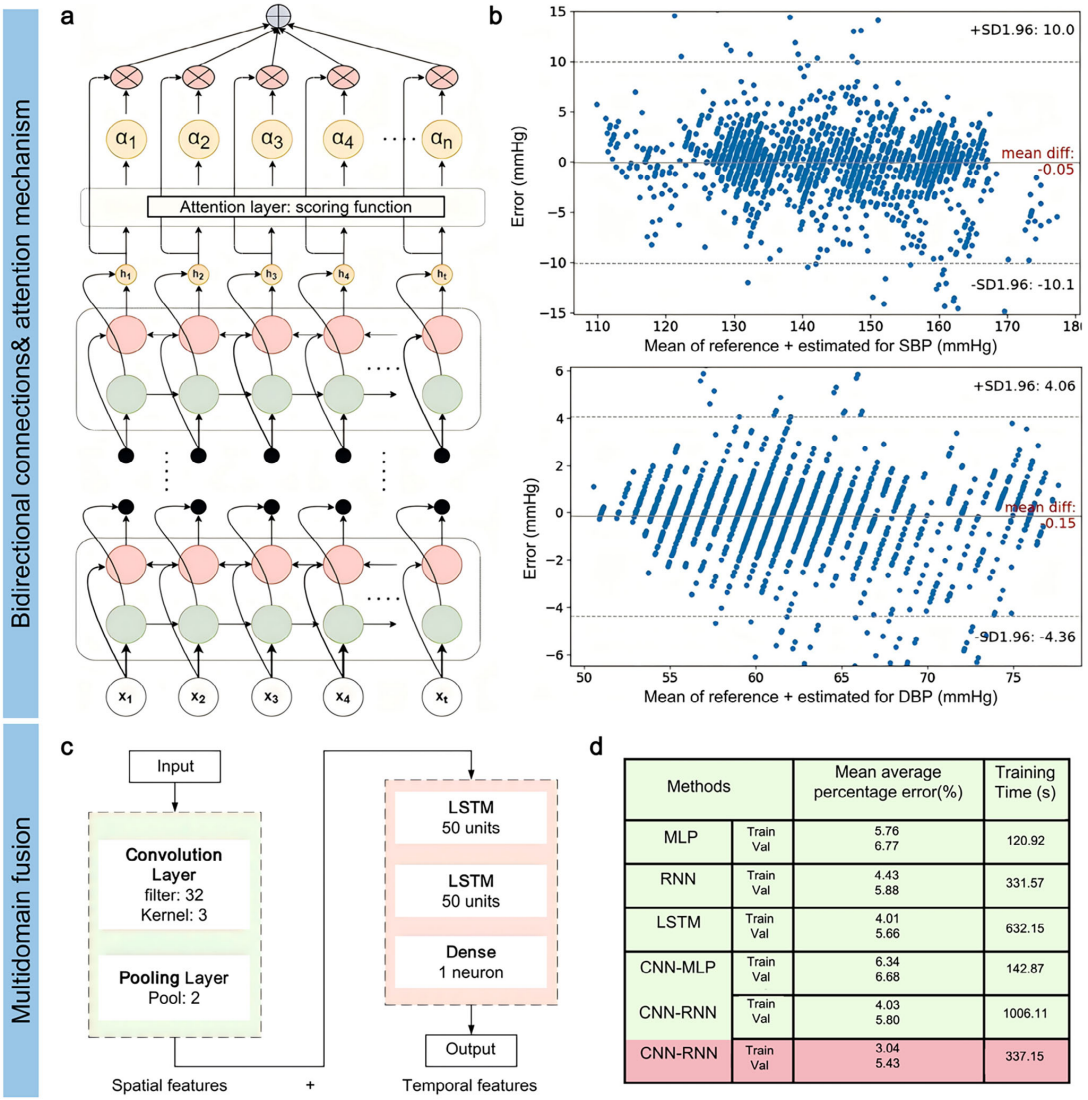
DL networks for different prediction tasks. a) Architecture of proposed method. Each dotted block represents a Bi‐RNN layer with forward (green) and backward (red) cells concatenated together (black dot) after each block. The output of the last Bi‐RNN block is passed through the attention layer to calculate the weights *α* and multiply it by its corresponding hidden state *h*. The results are then summed up, and SBP and DBP values are calculated. b) As estimated by Bi‐GRU, bland‐Altman plots for SBP (left) and DBP (right) are with attention. Reproduced with permission.^[^ ^230^ ^]^ Copyright 2021, Elsevier. c) The topology of the CNN‐LSTM model. d) The predicted performance of the proposed method with significantly reduced training time. Reproduced with permission.^[^ ^209^ ^]^ Copyright 2021, MDPI (Basel, Switzerland).

Similarly, El‐Hajj et al. introduced multiple systolic and diastolic BP estimation models based on RNNs with bidirectional connections and an attention mechanism utilizing PPG signals.^[^ ^230^ ^]^ Conventional LSTM and GRU networks aim to capture information from the input data processed through their hidden state, incorporating history and the current input observation. Due to their unidirectional nature, these networks can only retain information from previous time steps. To enhance the network's context and improve accuracy, bidirectional RNN (Bi‐RNN) connections can be employed. Bi‐RNNs incorporate past, present, and near‐future information from the input sequence, where all time steps are available. This paper presents two distinct architectures for this purpose. The first architecture, illustrated in **Figure** **14**a, comprises stacked Bi‐RNN layers followed by an attention layer. In this study, the traditional RNN units are replaced by LSTM and GRU units, which are tested separately for each proposed architecture. The models were evaluated on two datasets: one containing 22 PPG features and another comprising seven features identified as most effective for blood pressure estimation. Subsequently, various dimensionality reduction techniques were applied to eliminate redundant information and minimize computational complexity. The best accuracy on the 7 feature set was 2.9 ± 3.94 mmHg and 1.31 ± 1.76 mmHg for SBP and DBP, respectively, as shown in Figure 14b. The results of these studies underscore the potential of deep learning in accurately estimating BP values, which can revolutionize the field of cardiovascular health monitoring. Additionally, Slapničar et al. utilized data from 510 subjects, employing a spectro‐temporal ResNet deep learning model to model the dependency between PPG and BP, achieving mean absolute errors of 9.43 mmHg for SBP and 6.88 mmHg for DBP.^[^ ^231^ ^]^

However, the performance of models relying on a single feature domain is often limited. Integrating multi‐domain techniques to analyze pulse waveforms can enhance blood pressure prediction. For instance, a hybrid model may employ a CNN to extract spatial features from the waveform, while an RNN captures temporal dependencies. This combination of DL and signal processing enables a comprehensive analysis of pulse waveforms. Mou et al. proposed a CNN‐LSTM hybrid model for blood pressure prediction based on pulse waveform data,^[^ ^209^ ^]^ as illustrated in Figure 14c. In this approach, CNN extracts spatial features from the pulse waveform data, which are input into the LSTM to get temporal features for further training. Numerical results in Figure 14d from real‐life datasets demonstrate that this method achieves high predictive blood pressure accuracy while reducing training time.

Moreover, generative models, such as Generative Adversarial Networks (GANs) and Variational Autoencoders (VAEs), are increasingly employed in unsupervised learning to generate synthetic data, including pulse waveforms.^[^ ^232^, ^233^, ^234^ ^]^ These models can be trained on real datasets to capture the underlying distribution of the waveforms, enabling the generation of new synthetic pulse waveforms that closely resemble actual ones. Such synthetic data can be utilized for various applications, including data augmentation, anomaly detection, and the creation of virtual patient data for simulation purposes.

**Figure 15:**
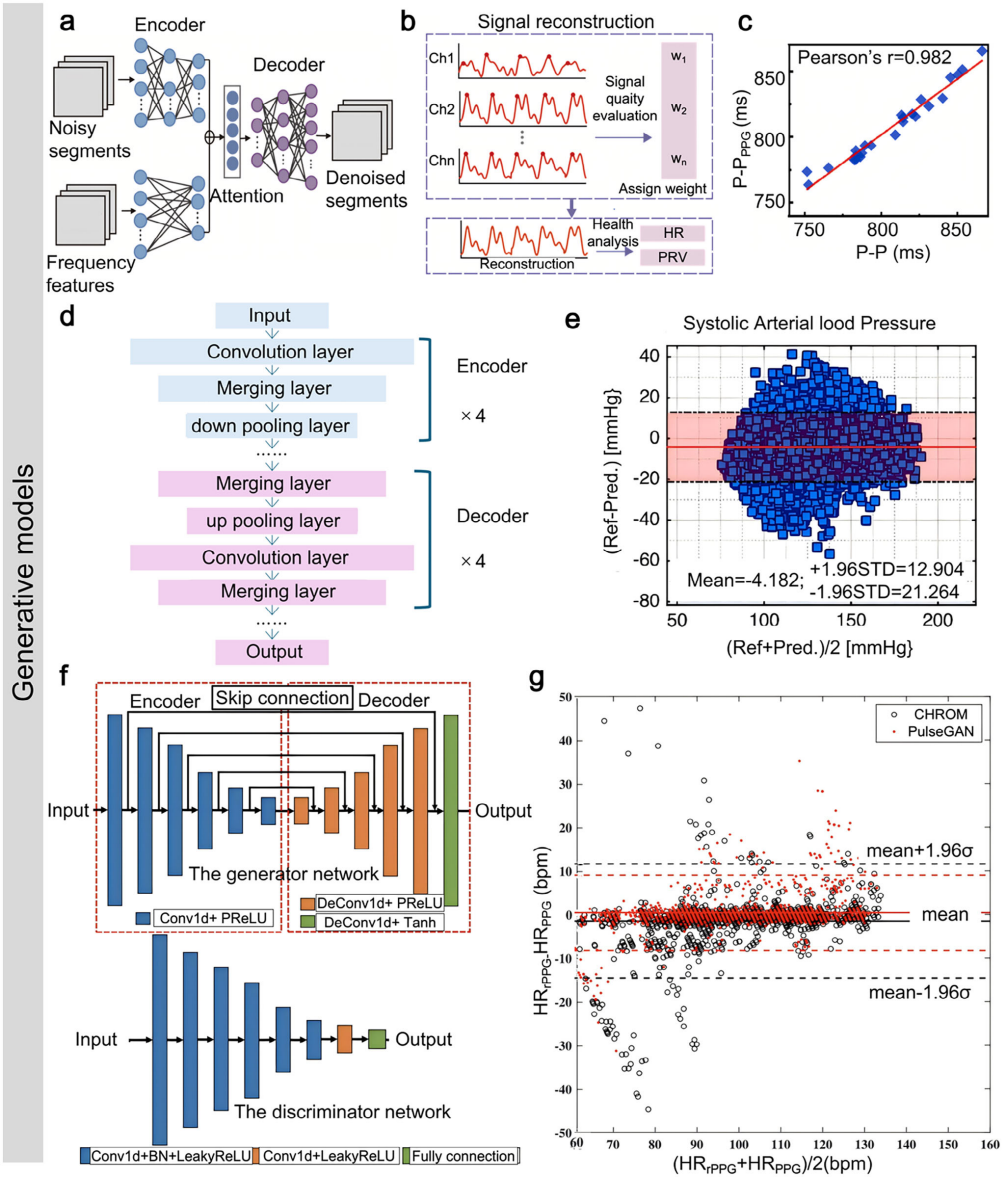
The DL methods for pulse waveforms. a) Schematic diagram of autoencoder model for signal enhancement. b) Schematic diagram of Inverse‐variance weighting algorithm for pulse signal reconstruction. c) The Pearson correlation plot of beat‐to‐beat and PPG pulse peak intervals. Reproduced with permission.^[^ ^232^ ^]^ Copyright 2024, Elsevier. d) Cardiovascular hemodynamics MA‐UDCAE model structure. e) The Bland‐Altman plot of both SABP and DABP. Reproduced with permission.^[^ ^233^ ^]^ Copyright 2021, MDPI (Basel, Switzerland). f) Network structure of PulseGAN. g) Bland‐Altman plots the difference between the predicted and reference HR for a cross‐database. Reproduced with permission.^[^ ^234^ ^]^ Copyright 2021, IEEE.

Jia et al. developed a deep learning model based on a denoising autoencoder (DAE) and an attention mechanism to reduce noise and improve the quality of pulse signals.^[^ ^232^ ^]^ The DAE model consists of three main parts: encoder, attention module, and decoder. The input consists of two streams: a noisy signal segment and its frequency characteristics. The three dense layers process noisy signals, while the two dense layers process frequency features. These two input streams are then compressed into the potential space of the encoder, and the fusion of time and frequency features is achieved through the attention module, as depicted in **Figure** **15**a. Finally, the decoder reconstructs the underlying data representation into a de‐noised signal, as illustrated in Figure 15b. A pulse sensor measured the pulse intervals calculated from the signal, and they were highly correlated with PPG pulse intervals, with a Pearson's correlated coefficient of 0.982, as shown in Figure 15c, demonstrating its substitutability for commercial devices. This reported technology suggests a novel approach for pulse waveform detection in static and dynamic conditions, allowing users to achieve reliable pulse monitoring easily. For BP prediction, Muammar et al. investigated the generation of continuous arterial blood pressure (ABP), central venous pressure (CVP), and PAP using a multi‐atrous U‐Net‐based deep convolutional autoencoder (MA‐UDCAE), as depicted in Figure 15d, utilizing only a single ECG sensor.^[^ ^233^ ^]^ The results in Figure 15e demonstrated high reliability, with correlation coefficients of 0.894 ± 0.004 mmHg for SBP and 0.881 ± 0.005 mmHg for DBP, indicating the significant potential of ECG data in generating ABP, CVP, and PAP waveforms, along with their essential information for comprehensive evaluations.

Additionally, Song et al. introduced a novel framework based on GANs, termed PulseGAN, illustrated in Figure 15f, which aims to generate realistic remote rPPG pulse signals by denoising chrominance (CHROM) signals.^[^ ^234^ ^]^ The effectiveness of the proposed framework was evaluated across three public databases, with results showing that the PulseGAN framework significantly improved waveform quality, as shown in Figure 15g, thereby enhancing the accuracy of HR, interbeat interval (IBI), and related heart rate variability (HRV) features.

However, the rapid advancement of DL still needs to render traditional ML methods obsolete. Paiva et al. developed an automated approach utilizing supervised learning techniques to differentiate between healthy and pathological arterial pulse waveforms and identify noisy waveforms using data acquired during clinical examinations with a novel optical system.^[^ ^214^ ^]^ The signals were characterized by 39 pulse features, including morphological attributes, time‐domain statistics, cross‐correlation, and wavelet features. Their findings indicated that SVM outperformed artificial neural networks (ANN) in this context, achieving an accuracy exceeding 99%. This underscores that the choice of ML algorithm is contingent upon several factors, including the specific nature of the problem (e.g., regression, classification, or clustering), data size, data distribution, feature relationships, and the assumptions and properties inherent to the algorithm. Furthermore, considerations regarding prediction accuracy and computational efficiency are also crucial in the selection process.

Signal processing technology grounded in hemodynamics enhances wearable devices’ accuracy, reliability, and usability for monitoring cardiovascular health. Through signal acquisition, preprocessing, and feature extraction, this technology facilitates precise measurements, real‐time monitoring, personalized healthcare, remote surveillance, data‐driven insights, and seamless integration with wearable devices. With continued advancements, it holds substantial promise for improving healthcare outcomes and fostering a proactive approach to cardiovascular wellness.

### 6.3 Artificial Intelligence‐Assisted Flexible Pulse Sensors for CVD Management

Artificial intelligence (AI)–assisted flexible pulse sensors have emerged as a promising technology for managing CVD by facilitating continuous monitoring, early diagnostics, and personalized healthcare. Various intelligent, flexible pulse sensors have been developed for hemodynamics monitoring and enhanced CVD care in this context.^[^ ^235^ ^]^

**Figure 16:**
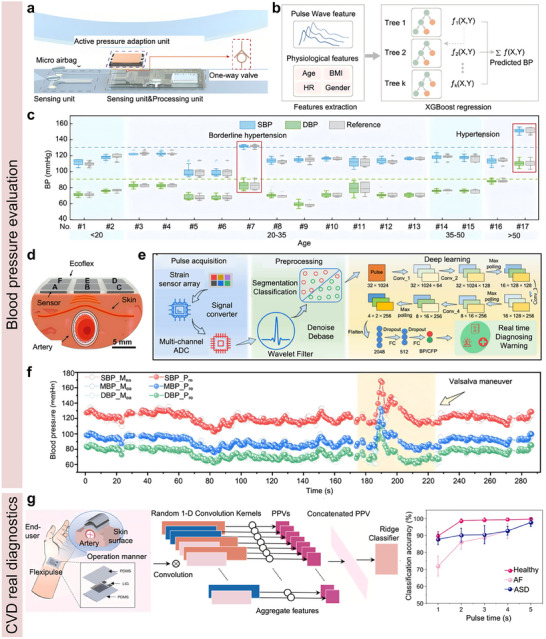
Artificial intelligence‐assisted flexible sensors for CVD diagnostics. a) A thin, flexible, wearable system (TSMS) based on piezoelectric response for continuous wireless monitoring of arterial BP. b) Illustration of the extracted pulse wave features, including systolic period (ST) and diastolic time (DT) and physiological features in the estimation model c) Statistical plots present the comparison between commercial device and proposed wristband with BioPAC system in SBP and DBP. Reproduced with permisssion.^[^ ^16^ ^]^ Copyright 2023, Springer Nature. d) An intelligent BP and cardiac function monitoring system based on six piezoresistive sensors e) The system flow chart contains pulse acquisition, preprocessing, and DL modules. f) Predicted BP via the proposed system and measured BP via a medical device in a continuous 5‐min test with a Valsalva maneuver event. Reproduced with permission.^[^ ^78^ ^]^ Copyright 2023, American Association for the Advancement of Science. g) A flexible and conformable pulse sensor with the architecture of ML model for disease diagnosis and the comparison of the classification accuracy achievable with various pulse wave times. Reproduced with permission.^[^ ^11^ ^]^ Copyright 2023, Elsevier.

When implementing a sensing system for realistic long‐term monitoring, ensuring the equipment's optimal fit and anti‐artifact capabilities is essential while considering the feedback mechanisms that facilitate a positive user experience. Yu et al. proposed a thin, flexible, wearable system (TSMS) based on piezoelectric response for the continuous wireless monitoring of ABP.^[^ ^16^ ^]^ This system comprises a conformal piezoelectric sensor array, an active pressure adaptation unit, a signal processing module, and an advanced ML method, as illustrated in **Figure** **16**a. Encapsulated in silicone and designed in a wristband format, the TSMS incorporates an active pressure adaptation system that adjusts backpressure and employs a multi‐pumping control strategy to eliminate the mechanical stiffness associated with the PZT 5H sensor electrode, ensuring optimal contact between the sensor array and the skin. Additionally, the ML‐based data model incorporates a wireless communication strategy, allowing the measured blood vessel waveform to be preprocessed and used as input for the blood pressure estimation model, along with multiple extracted features and local PWV data, as depicted in Figure 16b. A slightly increasing trend can be found in BP with the increased age regardless of gender from both commercial device and this system, and excellent measurement robustness and accuracy, with 0.11 ± 3.68 mmHg for DBP and −0.05 ± 4.61 mmHg for SBP, was achieved by the TSMS, as shown in Figure 16c.

Beyond direct calculations of parameters such as HR and PWV, integrating artificial intelligence and big data technologies enables wearable sensing systems to contribute significantly to BP monitoring. Li et al. developed an intelligent BP and cardiac function monitoring system comprising pulse acquisition, preprocessing, and DL modules, utilizing six piezoresistive sensors.^[^ ^78^ ^]^ Their proprietary algorithms incorporate various preprocessing functions, including denoising, normalization, segmentation, and classification, structured around four convolutional layers, three max‐pooling layers, and two fully connected layers, as shown in Figure 16d. This architecture processes pulse data to calculate BP and cardiac function metrics, as illustrated in Figure 16e. The system employs carbonized silk georgette (CSG) with twisted warp and weft yarns as the active layer, while high‐conductivity, laser‐cut nickel fabric serves as the electrodes.

Ultrathin Ecoflex layers are utilized for encapsulation, providing excellent flexibility, biocompatibility, and structural and chemical stability. The system adheres stably and conformally to the skin, even during wrist flexion or twisting, and is aided by medical‐grade adhesive to acquire high‐quality pulse wave signals. This comprehensive system offers detailed insights into cardiac status, including key parameters such as DBP, SBP, MBP, pulse pressure variation (PPV), CO, ejection fraction (SV), and systemic vascular resistance (SVR). The predicted BP patterns are then transferred to a mobile graphical user interface (GUI), where the user's continuous blood pressure patterns are displayed and recorded for further analysis. Predicted BP via the proposed system and measured BP via a medical device in a continuous 5‐min test with a Valsalva maneuver event, as illustrated in Figure 16f.

In addition to their monitoring capabilities, these devices are expected to serve as auxiliary tools for clinical diagnosis, potentially reducing the burden on healthcare systems. Flexible wearable sensors are increasingly being employed to diagnose specific CVD conditions. For instance, Ma et al. developed a flexible and conformable intelligent pulse sensor, FlexiPulse, fabricated from porous graphene using an eco‐friendly laser direct‐engraving technique,^[^ ^11^ ^]^ as shown in Figure 16g. The ultra‐thin design of FlexiPulse allows for exceptional flexibility, enabling it to self‐adhere to human skin without needing external pressure. The sensor exhibits a high gauge factor of 2336 (with a strain of 2.75–3%), an ultralow detection limit of 0.0056%, and remarkable stability over more than 24 000 cycles. Subsequently, clinical tests were conducted involving actual CVD events, encompassing healthy subjects, atrial fibrillation (AF), and atrial septal defect (ASD), to collect continuous pulse wave data. Drawing inspiration from traditional Chinese medicine, the researchers developed ML algorithms to classify these CVD events, achieving an impressive average accuracy of 98.7%.

AI‐assisted flexible sensors enable continuous monitoring of vital signs and cardiovascular parameters, facilitating the early detection of potential cardiac events and enhancing personalized diagnostics. These sensors can be seamlessly integrated into wearable devices or smartphone applications, empowering individuals to manage their cardiovascular health actively. The capability for remote patient monitoring and data transmission to healthcare providers supports proactive interventions and the development of personalized treatment plans. As technology continues to evolve, the integration of AI and flexible sensors promises to transform the field of cardiovascular disease diagnostics, ultimately leading to improved management and prevention of cardiovascular conditions.

## 7 Conclusions and Perspectives

This review examined the latest developments in bio‐integrated flexible electronics for hemodynamic monitoring, emphasizing their transformative potential in cardiovascular healthcare. While substantial advancements have been achieved, these technologies still face critical challenges that must be addressed to facilitate their practical and clinical applications in intelligent cardiovascular care.

Enhancing the sensitivity of bio‐integrated flexible electronics is paramount to meet the rigorous standards required for clinical‐grade devices. This enhancement is essential for accurately detecting subtle hemodynamic variations, ultimately improving diagnostic precision. Achieving this goal necessitates innovative approaches in materials design, selecting advanced sensing mechanisms, and optimizing mechanical structures and fabrication strategies.^[^ ^236^ ^]^ Furthermore, the performance of hemodynamic monitoring systems must remain stable under various challenging conditions, including mechanically deformed skin and fluctuating hygrothermal environments. Fusing multiple sensing modalities is a promising avenue to provide comprehensive data that enhances diagnostic accuracy and clinical utility.

In addition, developing high‐integration systems is necessary for the evolution of bio‐integrated flexible electronics. Future devices should seamlessly incorporate flexible sensors, wireless communication modules, embedded algorithms, and alert systems into a fully autonomous and user‐friendly platform. Such holistic designs improve usability and enable real‐time monitoring capabilities, which are vital for effective healthcare delivery. Future research should focus on several key aspects to fully harness the capabilities of bio‐integrated flexible electronics. First, there is a pressing need to develop sustainable, biocompatible, and potentially biodegradable materials, especially for invasive applications. This approach enhances patient safety and addresses growing environmental concerns associated with medical waste. Second, the accuracy of hemodynamic monitoring must consistently align with established clinical standards; this necessitates rigorous validation through extensive clinical trials to confirm the reliability and stability of these devices for practical use.

Integrating AI within bio‐integrated electronics is a significant frontier for advancing real‐time data processing and intelligent decision‐making, particularly in monitoring and managing cardiovascular disease. However, implementing AI must consider the limitations of low‐power operation, especially with small, flexible devices. Establishing secure and efficient data‐sharing frameworks through cloud and edge computing is essential for ensuring patient data privacy while providing scalable solutions for healthcare providers. Advanced algorithms for data analysis must be tailored to operate effectively with minimal sample sizes, maintaining high precision and rapid response times. This includes designing self‐calibrating systems that can identify faults and adapt to individual user profiles, enhancing the personalization of monitoring solutions. Furthermore, exploring dynamic physiological monitoring systems capable of long‐term data collection and validation in clinical environments is crucial, emphasizing improving user satisfaction through intuitive mobile interfaces and effective integration within IoT frameworks. While significant hurdles exist to widespread clinical adoption, synergizing bioelectronics with AI can fundamentally reshape healthcare delivery.

Finally, achieving these ambitious goals will demand a robust interdisciplinary approach synthesizing insights from biomedical engineering, materials science, data science, and clinical medicine. By fostering collaboration across these fields, we can advance the development of high‐performance bio‐integrated flexible electronics, ultimately paving the way for innovative solutions in intelligent healthcare.

## Conflict of Interest

The authors declare no conflict of interest.

## Author Contributions

K.H. and Z.M. contributed equally to this work. Z.M. and B.K. conceived the idea and designed and supervised the projects. K.H., Z.M., and B.K. wrote the manuscript. All authors reviewed and commented on the manuscript.
